# Bio-Inspired Controller Design via Dholes-Inspired Optimization: A Novel Gompertz Function-Augmented PID Strategy for Electro-Hydraulic Actuator Control

**DOI:** 10.3390/biomimetics11080535

**Published:** 2026-08-02

**Authors:** Muhammet İsmail Güngör, Davut Izci, Serdar Ekinci

**Affiliations:** 1 Department of Electrical and Electronic Engineering, Bursa Uludag University, Bursa 16059, Türkiye; muhammetismailgungor@uludag.edu.tr; 2 Department of Computer Engineering, Bitlis Eren University, Bitlis 13100, Türkiye

**Keywords:** PID augmented with a Gompertz function, hydraulic control system, dholes-inspired optimization, stability, closed-loop response

## Abstract

Electro-hydraulic actuator systems are widely used in precision motion-control applications; however, their displacement regulation remains challenging because fast response, low overshoot, and high steady-state accuracy must be achieved simultaneously under strongly dynamic operating conditions. In this study, a proportional-integral-derivative (PID) controller augmented with a Gompertz function (PID-G) is proposed for the position control of a four-way valve-controlled linear actuator, and its parameters are tuned by the recently introduced dholes-inspired optimizer (DIO). First, a control-oriented mathematical model of the electro-hydraulic actuator system is established by combining the valve and actuator dynamics. Then, the PID-G structure is formulated by incorporating a nonlinear Gompertz-based term into the conventional PID framework, and the resulting seven-parameter tuning problem is cast as an optimization task using a composite objective function that accounts for overshoot, steady-state error, rise time, and settling time. The effectiveness of DIO is evaluated comparatively against flood algorithm (FLA), covariance matrix adaptation evolution strategy (CMA-ES), and particle swarm optimization (PSO) under identical simulation conditions. The results show that DIO provides the best optimization performance, yielding the lowest best, average, and standard-deviation values of the objective function among the compared algorithms. In the time domain, the DIO-based PID-G controller achieves the most favorable overall response with a rise time of 0.079511 s, a settling time of 0.099326 s, an overshoot of 0.15110%, and a steady-state error of 0.089317%. The superiority of the DIO-based design is further confirmed by lower values of error based performance metrics (IAE, ISE, ITAE, and ITSE), improved convergence characteristics, and statistically significant advantages in the Wilcoxon test. Additional comparisons with different (PI, PID, 2DOF-PID, and FOPID) controllers also demonstrate that the proposed PID-G structure provides markedly better transient and error-based performance when tuned by DIO. Frequency-domain and varying-setpoint results further indicate satisfactory stability margins, robust tracking ability, and bounded control effort. Overall, the study shows that combining DIO with a Gompertz-augmented PID structure constitutes an effective strategy for high-performance electro-hydraulic actuator displacement control.

## 1. Introduction

Electro-hydraulic actuation systems occupy a central place in industrial motion-control applications because they combine high force density, fast dynamic response, and practical suitability for heavy-duty positioning tasks. For this reason, they have been used extensively in machine tools, construction and mining equipment, aerospace systems, testing platforms, and other applications where accurate displacement regulation must be achieved under demanding operating conditions. At the same time, the control of such systems remains challenging because valve-controlled hydraulic actuators exhibit strong nonlinearities, parameter sensitivity, flow–pressure coupling, friction effects, and dynamic interactions between hydraulic and mechanical subsystems. These characteristics make it difficult to obtain simultaneously fast rise, low overshoot, short settling time, and high steady-state accuracy by means of a straightforward linear design [1,2,3].

Within this context, the displacement control of a four-way valve-controlled linear actuator represents a practically important and technically nontrivial problem [4]. In such systems, actuator motion is governed through spool-valve dynamics and chamber pressure variations, and the resulting closed-loop behavior depends strongly on both plant dynamics and controller tuning. Even when classical feedback structures are employed, satisfactory performance may not be obtained unless the controller parameters are selected carefully. This difficulty becomes more pronounced when the design objective is not limited to a single performance index, but instead requires a balanced compromise among transient speed, overshoot suppression, and steady-state precision. The present manuscript addresses this problem by focusing on the position control of a four-way electro-hydraulic-valve-controlled linear actuator and by formulating the controller tuning task as a multi-parameter optimization problem.

Because of their simplicity, interpretability, and proven industrial utility, proportional-integral-derivative (PID)-family controllers [5,6] continue to be widely adopted in hydraulic and electro-hydraulic applications. Nevertheless, the limitations of conventional PID regulation have also been emphasized repeatedly, especially when strong nonlinearities, uncertainties, and demanding transient specifications are present. Fallahi et al. proposed a robust linear approximation framework for precise electro-hydraulic servo positioning and showed that accurate control can be improved through better handling of model nonlinearities [1]. Nguyen et al. developed an adaptive robust position-control strategy for electro-hydraulic servo systems under large uncertainties and disturbances, demonstrating the continuing need for advanced control formulations in this class of systems [2]. Likewise, Zaare and Soltanpour introduced an optimal robust adaptive fuzzy backstepping approach for electro-hydraulic servo position systems, highlighting the importance of uncertainty compensation and nonlinear design mechanisms [3]. These studies confirm that electro-hydraulic position regulation remains an active research field in which classical linear designs are often insufficient when higher performance is required.

In parallel with robust and adaptive schemes, several studies have examined how PID-based structures can be improved either through enhanced tuning or through structural extensions. Feng et al. optimized PID coefficients for an electro-hydraulic position servo system by means of an improved PSO-based strategy while accounting for uncertain nonlinear characteristics, and reported improved trajectory tracking performance [7]. Guo et al. investigated Kalman-genetic-optimization-based PID position control for a hydraulic servo system and showed that combining classical PID control with optimization and filtering can enhance dynamic response and anti-disturbance capability [8]. More recently, Liao et al. proposed a position-tracking strategy for an electro-hydraulic actuator system that combines a feedforward inverse model with an adaptive controller, again underscoring that stronger tracking performance often demands either structural enrichment or more capable tuning mechanisms [9]. Collectively, these studies indicate that the performance boundary of PID-type controllers is strongly influenced by how their parameters are tuned and by whether additional nonlinear or compensatory mechanisms are embedded into the control law.

A similar evolution can be observed in the wider PID literature. Joseph et al. provided a broad review of metaheuristic methods for PID parameter tuning and showed that optimization-based tuning has become a major direction for improving PID performance in nonlinear and multi-objective control problems [10]. At the same time, nonlinear PID structures have attracted increasing attention because they allow the control action to be shaped more flexibly than in conventional linear forms. This trend is reflected in the recent review by Çelik et al., where nonlinear PID formulations were discussed as an important pathway for improving control quality in complex dynamic systems [11]. In related work, sigmoid-based or otherwise nonlinear fractional/PID-type structures have also been explored in the literature, further supporting the view that nonlinear augmentation of PID controllers can provide additional design freedom beyond classical gain tuning [11].

Alongside controller-structure development, metaheuristic optimization [12] has assumed an increasingly important role in electro-hydraulic actuation systems, since the tuning of controller parameters in such systems is often complicated by nonlinear dynamics, hydraulic–mechanical coupling, uncertain characteristics, and the simultaneous need to satisfy multiple transient and steady-state objectives [13,14,15]. In the published literature, metaheuristic algorithms have been employed not only to improve classical PID tuning, but also to enhance more sophisticated control formulations for electro-hydraulic servo and actuator systems. For instance, improved particle swarm optimization has been used for trajectory control of electro-hydraulic position servo systems, Kalman-genetic optimization has been incorporated into PID position control of hydraulic servo systems, and particle-swarm-based feedforward PID frameworks have also been reported for electro-hydraulic servo applications in real-time hybrid testing [7,8,9]. Similarly, optimization-assisted designs have been explored for electro-hydraulic servo active suspension systems and for electro-hydrostatic actuator parameter tuning, further demonstrating that intelligent search strategies can provide an effective means of handling the high-dimensional and strongly coupled design space encountered in hydraulic actuation problems [10,11].

Considered together, the nonlinear extensions of PID control reported for electro-hydraulic actuator regulation fall broadly into two categories. The first relies on intelligent or rule-based compensation mechanisms, such as fuzzy-logic-assisted backstepping [3] or Kalman-assisted genetic tuning [8], where the nonlinear shaping of the control action is achieved through auxiliary inference structures or estimation layers; these approaches can improve disturbance handling and tracking accuracy, but typically introduce additional design burden in the form of rule-base construction, membership-function tuning, or estimator design, and often reduce the direct interpretability of the resulting control law relative to a standard PID structure. The second category retains the PID form itself but extends it through a fixed nonlinear or fractional-order operator, as surveyed by Çelik et al. [11], including sigmoid-type and fractional PID formulations; these preserve much of the simplicity of classical PID design but frequently introduce additional tuning parameters whose physical interpretation is not always straightforward, and, in the case of sigmoid functions with steep or discontinuous transitions, can risk abrupt changes in the control signal near the operating point. The Gompertz-based augmentation proposed in this study belongs to this second category but is distinguished by combining a smooth, continuously differentiable, and analytically bounded nonlinear transition, governed by only three interpretable shape parameters, with a control action that saturates gradually rather than switching abruptly, thereby avoiding both the auxiliary design burden associated with rule-based or estimator-driven schemes and the abrupt transition behavior that can arise in steeper sigmoid-type nonlinear PID formulations.

Despite this substantial body of work, an identifiable research gap remains. Existing studies on electro-hydraulic actuator control have largely focused on robust linear approximation, adaptive robust control, fuzzy backstepping, improved PID tuning, and fractional or fuzzy FOPID formulations [1,2,3,7,12]. Although these contributions have advanced the state of the art, the available literature still shows limited attention to nonlinear PID architectures in which the additional control action is generated explicitly through a Gompertz-type function [16], originally formulated for growth-curve modelling, and subsequently embedded directly into the controller structure for electro-hydraulic displacement regulation—a class of problems that has instead been addressed predominantly through alternative nonlinear or intelligent control structures [17,18,19]. Moreover, while a variety of optimization algorithms have already been adopted for hydraulic servo and actuator tuning, the recently introduced dholes-inspired optimizer (DIO) [20] has not, to the best of the author’s knowledge, been reported for the parameter tuning of a four-way valve-controlled electro-hydraulic linear actuator. A further limitation is that many reported studies emphasize either controller design or optimization performance alone, whereas no studies provide a unified framework in which a newly introduced nonlinear controller structure is examined through objective-function minimization, statistical analysis, convergence behavior, time-domain evaluation, error-based criteria, benchmarking against alternative PID variants, frequency-domain assessment, and varying-setpoint tracking. It is precisely this gap that motivates the present study.

Motivated by these considerations, a proportional–integral–derivative controller augmented with a Gompertz function, denoted as the PID-G controller, is proposed in this study for the displacement control of a four-way valve-controlled electro-hydraulic linear actuator. In the proposed structure, the conventional proportional, integral, and derivative actions are complemented by an additional nonlinear branch generated through the Gompertz function [21], while the derivative term is implemented with first-order filtering to improve practical realizability. Owing to this formulation, greater flexibility is introduced into the control law, allowing the transient response to be shaped more effectively without sacrificing smoothness near the reference point. Since the resulting controller contains seven adjustable parameters, the tuning task is formulated as a constrained optimization problem and solved by means of the dholes-inspired optimizer (DIO) [20]. To obtain a balanced closed-loop response, a composite objective function is adopted in which overshoot, steady-state error, rise time, and settling time are considered simultaneously. To the best of the authors’ knowledge, this study presents the first reported implementation of a Gompertz-augmented PID controller for a four-way electro-hydraulic linear actuator, and also the first application of DIO to this specific controller-design problem.

The effectiveness of the proposed design is examined through a comprehensive simulation framework rather than through a single response comparison. In addition to objective-function minimization analysis, the obtained results are evaluated statistically over multiple independent runs by means of the Wilcoxon test [22], and the convergence characteristics of the competing algorithms are also examined. Closed-loop performance is further assessed through time-domain response analysis and through the integral absolute error (IAE) [23], integral squared error (ISE) [24], integral time absolute error (ITAE) [25], and integral time squared error (ITSE) [26] criteria. Moreover, the practical value of the proposed controller structure is clarified by benchmarking it against PID [15], two-degree-of-freedom PID (2DOF-PID) [27], and fractional-order PID (FOPID) [28] controllers tuned under the same optimization framework. Frequency-domain analysis by means of the Bode plot, together with varying-setpoint tracking and controller-output examination, is also carried out in order to provide a broader evaluation of the proposed method. The reported results show that the DIO-based PID-G controller yields the most favorable overall performance among the compared approaches. In particular, a rise time of 0.079511 s, a settling time of 0.099326 s, an overshoot of 0.15110%, and a steady-state error of 0.089317% are achieved, while the lowest values are also obtained for the considered error-based performance indices. These findings indicate that the proposed design provides not only improved optimization performance, but also a well-balanced and technically effective closed-loop response for electro-hydraulic actuator displacement regulation.

The main contributions of the present study may therefore be summarized as follows:

1. A new nonlinear controller architecture is introduced by augmenting the classical PID structure with a Gompertz-function-based term for four-way electro-hydraulic linear-actuator displacement control.
2. The associated seven-parameter tuning problem is formulated as a constrained optimization task and solved by means of DIO, which, to the best of the authors’ knowledge, has not previously been applied to this specific controller-design problem.
3. The effectiveness of this optimization strategy is assessed comparatively against the flood algorithm [29], covariance matrix adaptation evolution strategy [30], and particle swarm optimization [31] under identical simulation conditions.
4. The practical merit of the proposed PID-G controller is demonstrated through additional comparisons with PI, PID, 2DOF-PID, and FOPID structures, all optimized within the same DIO framework, thereby isolating the structural contribution of the Gompertz augmentation.
5. The proposed method is supported through complementary time-domain, error-based, frequency-domain, and varying-setpoint analyses, together with a dedicated discussion of implementation feasibility and practical considerations, thereby providing a comprehensive and multi-perspective assessment of its control performance.

The remainder of the paper is organized as follows: Section 2 introduces the dholes-inspired optimizer, describing its biological inspiration, mathematical formulation, and iterative search procedure. Section 3 presents the mathematical modeling of the four-way electro-hydraulic-valve-controlled linear actuator, deriving the governing equations and the numerical plant transfer function used throughout the study. Section 4 develops the proposed PID-G controller structure, defines the complete controller output equation and filtered derivative formulation, and positions the Gompertz-based nonlinear augmentation explicitly against sigmoid-based and adaptive nonlinear PID alternatives. Section 5 formulates the optimization problem, defines the composite objective function, specifies the controller parameter search bounds, and describes the application of DIO to the controller tuning task. Section 6 presents the simulation results across nine complementary subsections, covering objective function minimization performance, statistical analysis, convergence behavior and best parameter identification, time-domain evaluation, error-based performance assessment, benchmarking against alternative PID-type controller variants, frequency-domain stability analysis, setpoint-tracking and controller-output behavior, and a dedicated discussion of implementation feasibility and practical considerations. Finally, Section 7 concludes the paper with a summary of the principal findings, a balanced assessment of the advantages and limitations of the proposed method, and directions for future research.

## 2. Dholes-Inspired Optimizer

The dholes-inspired optimization (DIO) is a recently proposed population-based metaheuristic method designed by modeling the cooperative and adaptive hunting behavior of dholes, which are commonly known as Asiatic wild dogs [20]. Dholes exhibit highly organized social structures, in which pack members cooperate through vocal communication and coordinated movement during hunting. These behaviors enable the pack to effectively locate and capture prey within complex environments. Inspired by these natural mechanisms, the DIO translates these behaviors into computational strategies for solving nonlinear optimization problems. In the DIO framework, each candidate solution represents an individual dhole in the population. The collective population forms a hunting pack that searches for the optimal solution within the defined search space. The optimizer incorporates several biologically inspired mechanisms, including hierarchical leadership, adaptive communication via vocalization, coordinated cooperative hunting, and territorial constraints. These mechanisms allow the optimizer to dynamically balance global exploration and local exploitation while maintaining population diversity.

Let the population of candidate solutions be represented as $D=\{D_{1},D_{2},\ldots ,D_{N}\}$ where N denotes the population size and $D_{i}$ represents the position vector of the $i^{th}$ search agent in a d-dimensional search space. Initially, the positions of all search agents are randomly generated within the predefined lower and upper bounds of the problem as $D_{i}=lb+rand(1,d)\times (ub-lb)$ where lb and ub denote the lower and upper limits of the decision variables, respectively. After initialization, the fitness of each search agent is evaluated using the objective function of the optimization problem. The candidate solution with the best fitness value is selected as the lead vocalizer, which represents the most promising solution in the current iteration. This leader guides the search process and influences the movement of the remaining search agents.

One of the defining characteristics of dhole packs is their use of vocal communication to coordinate hunting movements. In the DIO, this behavior is modeled through a vocalization influence factor (vocal communication mechanism) that regulates the leader’s dominance during the search process. This factor gradually decreases as iterations progress to ensure a smooth transition from exploration to exploitation. The vocalization influence is defined as $V=2-t\left(2/Max\_iter\right)$ where V represents the vocalization influence coefficient, t denotes the current iteration number, and Max_iter is the maximum number of iterations. At the early stages of the search, the value of V remains large, encouraging exploration across wider regions of the search space. As iterations progress, the value decreases, thereby intensifying the exploitation of promising regions.

Dholes are well known for their cooperative hunting strategies, in which pack members adjust their positions dynamically in response to the leader and neighboring individuals. This coordinated behavior is translated into the DIO algorithm’s search mechanism via adaptive movement coefficients (cooperative hunting mechanism). A scaling factor, B=V×r, is introduced to regulate the movement intensity of search agents, where r is a uniformly distributed random number within the range [0,1]. To introduce controlled stochasticity into the search process and prevent premature convergence, an additional coefficient C is defined as C=r+sin(rπ). The sinusoidal component introduces oscillatory variations in the movement dynamics, enabling the search agents to explore new regions of the solution space while still being guided by the leader. Based on these coefficients, the adjusted distance between each search agent and the lead vocalizer is computed as follows [20]:

(1) $${\vec{D}}_{lead}=|C\times {{\vec{lead\_vocalizer}}_{pos}}^{2}-{{\vec{D}}_{i}}^{2}|$$

where ${\vec{lead\_vocalizer}}_{pos}$ denotes the position of the lead vocalizer and ${\vec{D}}_{i}$ represents the position of the $i^{th}$ search agent. The updated position of the search agent is then calculated as in Equation (2) [20]:

(2) $${\vec{X}}_{lead}={\vec{lead\_vocalizer}}_{pos}+B\times \sqrt{{\vec{D}}_{lead}}$$

This position update mechanism enables each search agent to move toward the leader while maintaining adaptive movement intensity, thereby preserving a balance between exploration and exploitation.

In natural environments, dhole packs exhibit territorial instincts that prevent individuals from straying too far from the group. Within the DIO, this behavior is represented through boundary constraints that ensure candidate solutions remain within feasible limits. If a search agent moves outside the allowable search space, its position is reinitialized within the bounds defined by Equation (3) [20]:

(3) $${\vec{D}}_{i}=\left\{\begin{array}{cc} lb+(ub-lb)\times rand(d), & \mathrm{if}\;{\vec{D}}_{i}<lb\;\mathrm{or}\;{\vec{D}}_{i}>ub\\ {\vec{D}}_{i}, & \mathrm{otherwise} \end{array}\right.$$

This boundary control mechanism prevents divergence of the search agents and ensures that all candidate solutions remain feasible throughout the optimization process.

At the end of each iteration, the fitness of all search agents is evaluated again. If a candidate solution achieves a better fitness value than the current leader, it replaces the lead vocalizer according to the following definition (leadership update) [20]:

(4) $$lead\_vocalizer=\left\{\begin{array}{ll} {\vec{D}}_{i},\;\mathrm{if}\;fitness({\vec{D}}_{i})<fitness(lead\_vocalizer) & \\ lead_{vocalizer},\;\mathrm{otherwise} & \end{array}\right.$$

This dynamic leadership mechanism ensures that the algorithm continuously follows the best solution found during the search.

**Figure 1:**
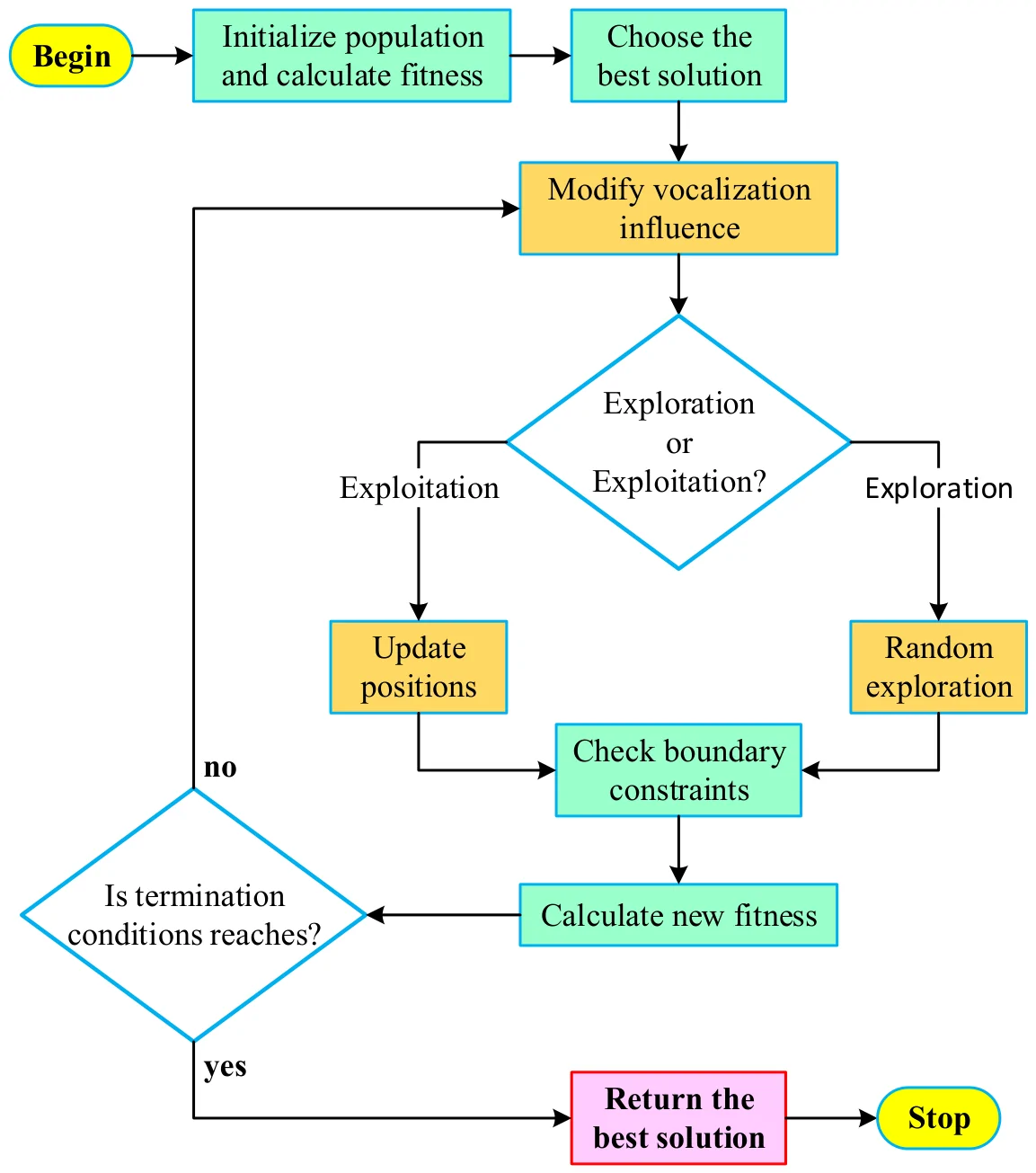
Flowchart of DIO.

The overall workflow of the DIO algorithm is illustrated in Figure 1. As shown in the flowchart, the optimization process begins with the random initialization of the dhole population and the evaluation of their fitness values. The best candidate solution is identified as the lead vocalizer, which guides the search process in subsequent iterations. The vocalization influence coefficient is then updated to regulate the balance between exploration and exploitation. Following this, a decision mechanism determines whether the algorithm should explore or exploit. During exploration, search agents randomly explore new regions of the search space. During exploitation, candidate solutions update their positions relative to the lead vocalizer using the cooperative hunting equations described earlier. After updating positions, boundary constraints are applied to ensure that the solutions remain within the feasible search region. The new fitness values of the updated solutions are then calculated. If the convergence criterion is satisfied, the algorithm terminates and returns the best solution obtained. Otherwise, the iterative process continues until the stopping condition is met. By integrating adaptive leadership, cooperative search dynamics, and boundary control mechanisms, the DIO algorithm provides a robust and efficient framework for solving complex optimization problems in engineering applications. The flowchart in Figure 1 summarizes these sequential steps and visually illustrates the interactions among exploration, exploitation, and solution evaluation during the optimization process.

## 3. Mathematical Modeling of Four-Way Electro-Hydraulic-Valve Control of Linear Actuator

**Figure 2:**
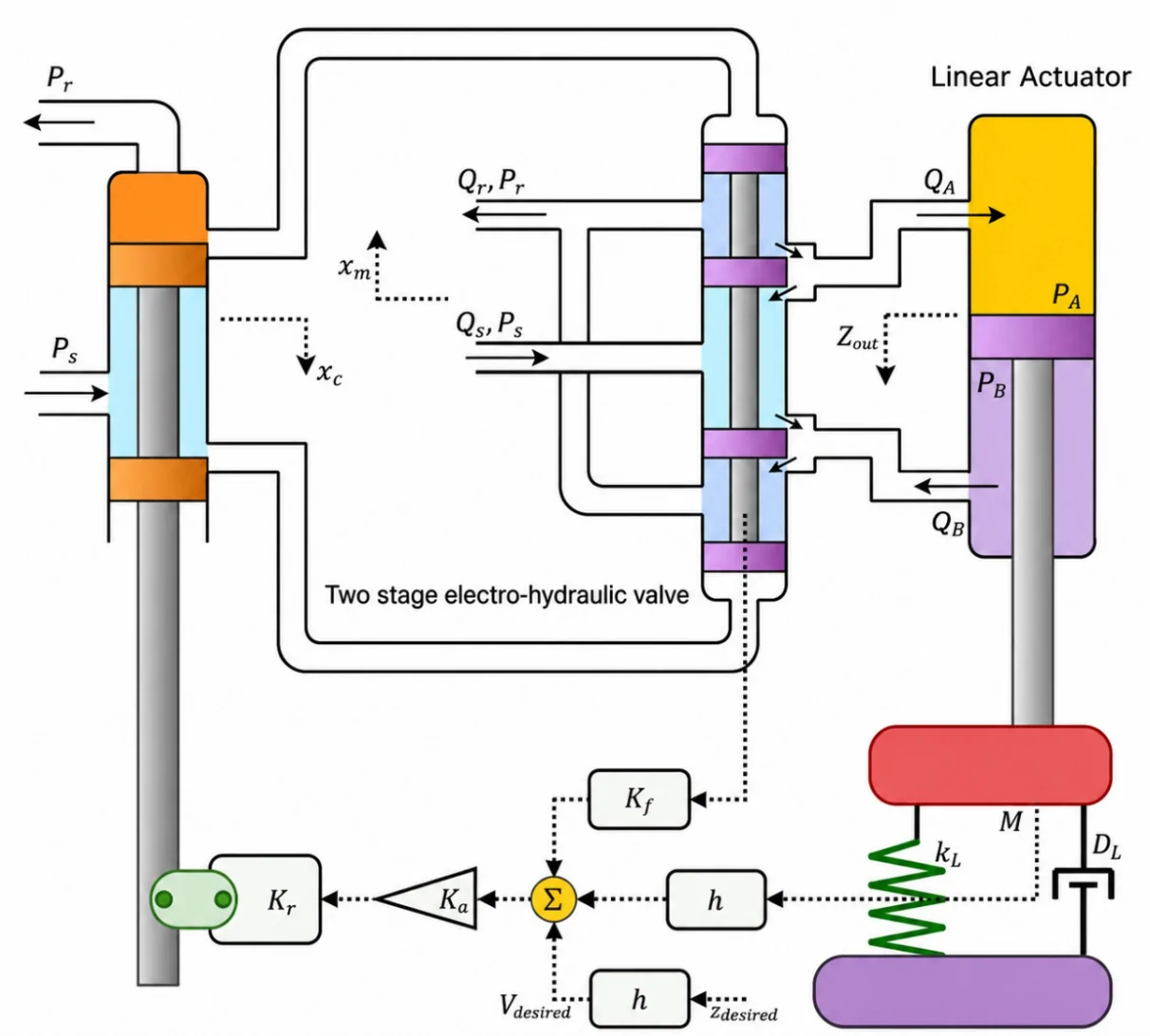
Schematic representation of a four-way valve-controlled linear actuator.

Electro-hydraulic actuation systems are widely employed in industrial motion-control applications because large output forces can be generated together with a relatively fast dynamic response. Among the commonly used hydraulic arrangements, the four-way valve-controlled linear actuator occupies an important place in precision positioning problems. In such a configuration, the actuator motion is governed by regulating the pressurized fluid delivered to the two actuator chambers through the spool-valve mechanism [32]. The arrangement considered in this study is illustrated in Figure 2, where a two-stage electro-hydraulic valve, a double-acting linear actuator, and the associated mechanical load are coupled in a closed-loop structure. As shown in the figure, the spool displacement $x_{m}$ determines the metering action of the valve, the chamber flow rates are represented by $Q_{A}$ and $Q_{B}$, and the actuator displacement is denoted by $z_{out}$. The external load is modeled by the mass M, damping $D_{L}$, and spring stiffness $k_{L}$. Accordingly, the actuator dynamics are established by linking valve flow, chamber pressure, and load motion in a systematic manner.

For the translational actuator, the rod force is generated by the pressure difference across the two sides of the piston. Under the ideal actuator assumption, the force balance may be written as follows [33]:

(5) $$F_{L}=A(P_{A}-P_{B})$$

where A is the piston area, while $P_{A}$ and $P_{B}$ denote the chamber pressures. Since the actuator displacement is represented by $z_{out}$, the volumetric flow into chamber A is obtained from the continuity relation as $Q_{A}=A{\dot{z}}_{out}$. The chamber flows are governed by the four-way spool valve.

For the critically centered operating condition, the linearized port-flow relations of the valve can be expressed as follows [33]:

(6) $$Q_{1}=-\frac{1}{2}Q_{0}-K_{q}x_{m}+K_{c}(P_{A}-P_{r})$$

(7) $$Q_{2}=\frac{1}{2}Q_{0}+K_{q}x_{m}+K_{c}(P_{s}-P_{A})$$

(8) $$Q_{3}=-\frac{1}{2}Q_{0}+K_{q}x_{m}+K_{c}(P_{s}-P_{B})$$

(9) $$Q_{4}=\frac{1}{2}Q_{0}-K_{q}x_{m}+K_{c}(P_{B}-P_{r})$$

in which $K_{q}$ is the flow gain of the control valve, $K_{c}$ is the pressure-flow coefficient, $P_{s}$ is the supply pressure, and $P_{r}$ is the return pressure. At the nominal operating point, the conditions $Q_{0}=0$ and $P_{r}=0$ are adopted, while the pressure levels on both sides of the actuator are centered about $P_{s}/2$. Using the net chamber-flow definitions $Q_{A}=Q_{2}-Q_{1}$ and $Q_{B}=Q_{4}-Q_{3}$, the actuator-side flow equations become as follows [33]:

(10) $$Q_{A}=2K_{q}x_{m}-2K_{c}\left(P_{A}-\frac{P_{s}}{2}\right)$$

(11) $$Q_{B}=2K_{q}x_{m}+2K_{c}\left(P_{B}-\frac{P_{s}}{2}\right)$$

By combining the continuity relation with the above flow expressions, the chamber pressures can be rearranged in terms of spool displacement and actuator velocity as follows [33]:

(12) $$P_{A}=\frac{K_{q}}{K_{c}}x_{m}-\frac{A}{2K_{c}}{\dot{z}}_{out}+\frac{P_{s}}{2}$$

(13) $$P_{B}=-\frac{K_{q}}{K_{c}}x_{m}+\frac{A}{2K_{c}}{\dot{z}}_{out}+\frac{P_{s}}{2}$$

These relations show that the pressure difference across the actuator is shaped jointly by the spool motion and the piston velocity. Substituting Equations (12) and (13) into the force relation of Equation (5), and then equating the actuator force to the mechanical load dynamics, yields the overall translational equation of motion as given in Equation (14) [33]:

(14) $$M{\ddot{z}}_{out}+\left(D_{L}+\frac{2A^{2}}{K_{c}}\right){\dot{z}}_{out}+k_{L}z_{out}=\frac{2AK_{q}}{K_{c}}x_{m}$$

Equation (14) indicates that the effective damping of the system is influenced not only by the physical load damping $D_{L}$, but also by the hydraulic term $2A^{2}/K_{c}$. Therefore, the spool-valve motion governs the actuator displacement through a coupled hydraulic-mechanical mechanism. Taking $x_{m}$ as the input and $z_{out}$ as the output, the actuator dynamics can be written in transfer-function form as given in Equation (15) [33]:

(15) $$\frac{Z_{out}(s)}{X_{m}(s)}=\frac{2AK_{q}}{MK_{c}s^{2}+(D_{L}K_{c}+2A^{2})s+K_{c}k_{L}}$$

The spool-valve itself is represented by a first-order characteristic. For the two-stage electro-hydraulic valve shown in Figure 2, the time constant is written as follows [33]:

(16) $$T_{c}=\frac{A_{m}}{K_{q}K_{a}K_{f}K_{r}}$$

where $A_{m}$ is the area of the main spool, $K_{a}$ is the proportional-controller gain associated with the valve stage, $K_{f}$ is the gain of the main spool feedback transducer, and $K_{r}$ is the ratio between the input voltage and the displacement of the control spool. Accordingly, the transfer function relating the valve error signal $V_{error}$ to the spool displacement $x_{m}$ becomes as given in Equation (17) [33]:

(17) $$\frac{X_{m}(s)}{V_{error}(s)}=\frac{1}{K_{f}(1+T_{c}s)}$$

By cascading the valve dynamics with the actuator dynamics, the control-oriented plant model used for the subsequent controller design is obtained, as given in Equation (18) [33]:

(18) $$G(s)=\frac{Z_{out}(s)}{V_{error}(s)}=\frac{2AK_{q}}{K_{f}(1+T_{c}s)\left[MK_{c}s^{2}+(D_{L}K_{c}+2A^{2})s+K_{c}k_{L}\right]}$$

The parameter values adopted in this study are summarized in Table 1 [33]. These values correspond to a representative four-way valve-controlled linear actuator and are used to define the plant (provided in Equation (19)) considered in this study. It is emphasized that the mathematical model presented in this section, including all governing equations from Equation (5) through Equation (18) and the associated system parameters listed in Table 1, is adopted directly from [31,32] without modification. The numerical plant transfer function given in Equation (19) is obtained by straightforward substitution of these parameter values into Equation (18) and is likewise derived entirely from the original model of [33]. The model is reproduced here solely to provide the analytical basis required for the controller synthesis and optimization procedures developed in the subsequent sections. It is emphasized that the contribution of the present study does not lie in this plant representation, which is adopted unmodified from established prior work, but in the nonlinear PID-G controller structure formulated in Section 4 and in the application of the dholes-inspired optimizer to its parameter tuning, as outlined in the Introduction. Adopting a previously validated plant model in this manner allows the reported performance improvements to be attributed specifically to the proposed controller structure and tuning strategy, rather than to differences in the underlying system representation.

(19) $$G\left(s\right)=\frac{3.033\times 10^{10}}{s^{3}+1.43\times 10^{5}s^{2}+9.295\times 10^{7}s+652.5}$$

**Figure 3:**
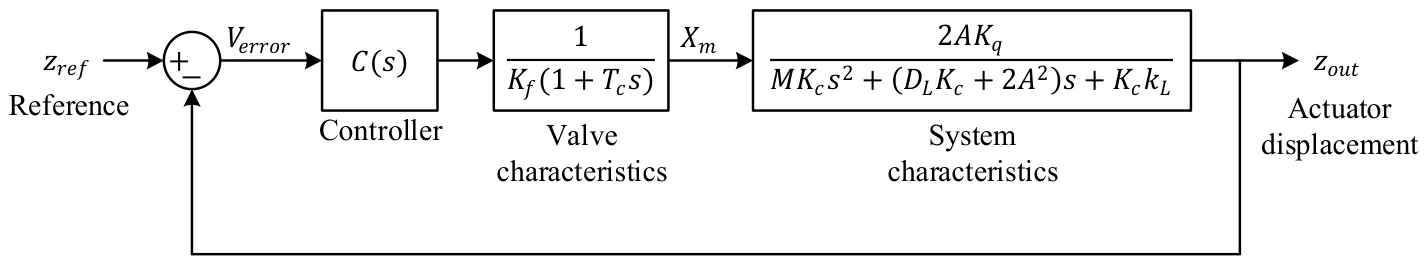
Position control of the four-way valve-controlled linear actuator.

The physical interpretation of the resulting model is summarized in Figure 3. As shown in the block representation, the reference displacement $z_{ref}$ is compared with the measured output to form the error signal $V_{error}$, which is then processed by the controller C(s). The controller output acts first on the valve block $1/[K_{f}(1+T_{c}s)]$, thereby determining the spool displacement $x_{m}$. This spool motion then excites the hydraulic-mechanical plant represented by Equation (15), whose output is the actuator displacement $z_{out}$. Hence, Figure 3 provides a compact control-oriented representation of the mathematical model, while Equations (5)–(19) furnish the analytical basis for the controller synthesis and optimization procedure developed in the following sections.

## 4. PID Augmented with a Gompertz Function Controller

In order to improve the displacement control performance of the four-way valve-controlled electro-hydraulic actuator, a PID structure [34] augmented with a Gompertz-type nonlinear term [35] is considered in this study. The motivation for employing the Gompertz function arises from its smooth sigmoidal characteristic [36], which enables a nonlinear control contribution to be introduced without causing abrupt variations in the control signal. In contrast to a purely linear PID structure, this formulation makes it possible to shape the control action in an adaptive nonlinear manner according to the instantaneous error magnitude. As a result, the controller can provide a stronger corrective effect in the transient region while still preserving smooth behavior as the tracking error approaches zero. The Gompertz function adopted in this work is given by Equation (20) [37]:

(20) $$f\left(t\right)=ae^{-be^{-ct}}$$

where a, b, and c are positive parameters governing the shape of the nonlinear function. In the proposed controller design, this function is not used as a time-domain growth model in its classical sense, but rather as a nonlinear mapping embedded into the control law through the tracking error. Owing to this structure, the Gompertz term can be tuned to modify the amplitude, horizontal shift, and growth rate of the nonlinear contribution in a flexible manner.

**Figure 4:**
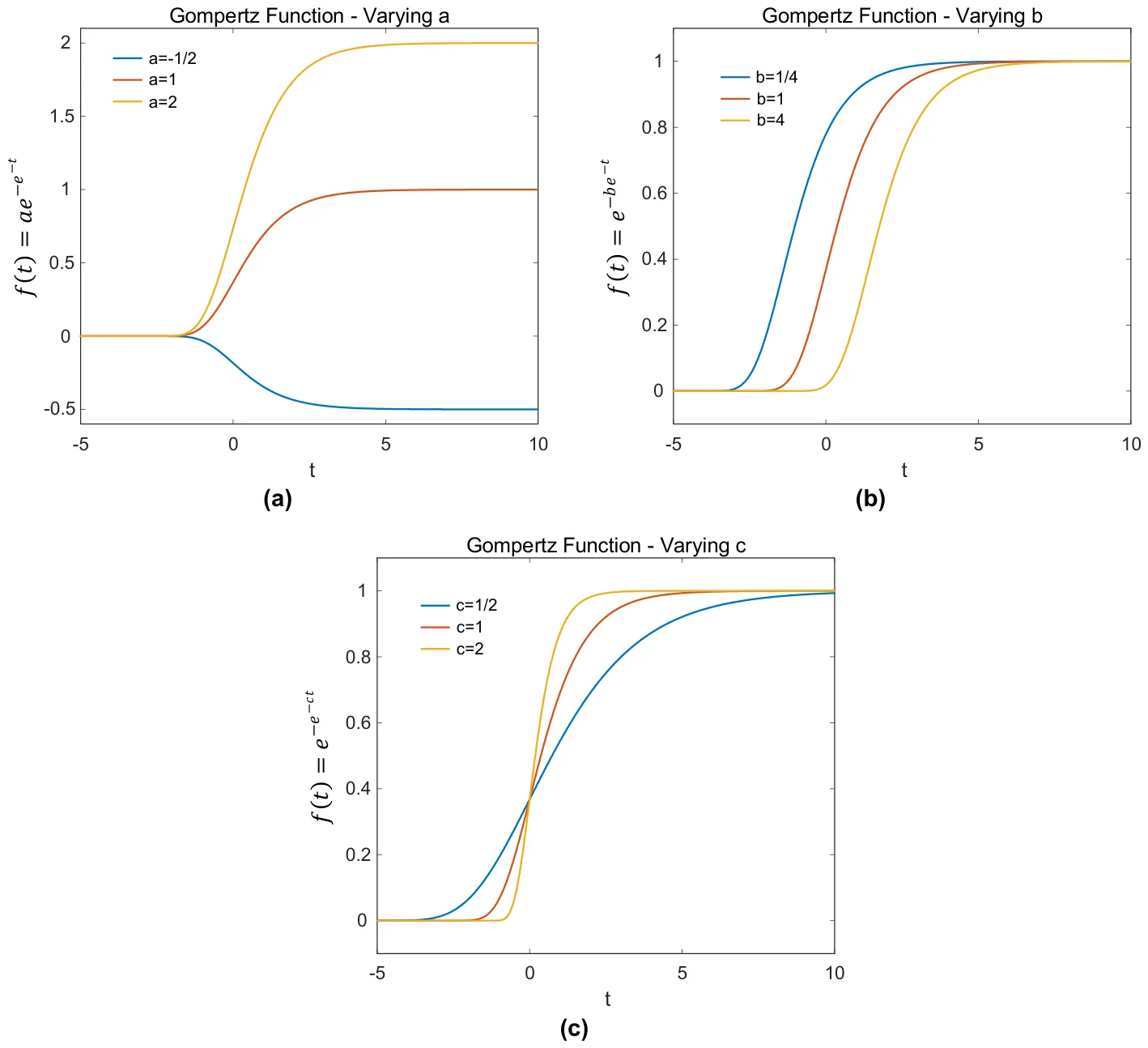
Gompertz function with respect to the effects of varying parameters a (**a**), b (**b**), and c (**c**).

The effects of the Gompertz parameters are illustrated in Figure 4. As shown in Figure 4a, the parameter a primarily determines the output level of the function and therefore controls the amplitude of the nonlinear contribution. An increase in a produces a higher asymptotic value, whereas a smaller a reduces the overall magnitude of the generated nonlinear signal. Figure 4b shows that the parameter b shifts the response along the horizontal axis, thereby changing the region in which the nonlinear contribution becomes effective. Hence, b influences the sensitivity of the function with respect to the input variable. Figure 4c demonstrates that the parameter c adjusts the rate of transition of the Gompertz curve. Larger values of c lead to a steeper change around the transition region, while smaller values yield a more gradual variation. Taken together, these three parameters provide sufficient flexibility for shaping the nonlinear action according to the control requirements of the electro-hydraulic actuator.

It is worthwhile to position the proposed use of the Gompertz function explicitly against sigmoid-based and adaptive nonlinear PID formulations that have been considered in the existing literature [11]. Sigmoid and hyperbolic tangent functions, which are commonly adopted as nonlinear activation elements in nonlinear PID designs, are symmetric about the origin and typically offer limited parametric flexibility (generally a single gain or slope parameter), making it difficult to independently control the amplitude, onset region, and rate of transition of the nonlinear contribution. The Gompertz function, by contrast, is inherently asymmetric: its double-exponential structure produces a distinctly skewed S-shaped curve in which the transition from the lower to the upper asymptote is markedly slower at small input values and comparatively faster near the inflection region.

This asymmetry is beneficial in displacement-control applications, where the control action needed during the initial approach to a setpoint differs substantially from the corrective action required near the steady-state point. Furthermore, the three independent shape parameters afford separate control over the amplitude, the horizontal activation threshold, and the steepness of the transition, respectively—a degree of design freedom that is not available in standard single-parameter sigmoid formulations. Regarding adaptive PID variants, such controllers typically require online identification or real-time gain scheduling based on plant state or model estimates, which introduces additional computational overhead and raises practical concerns related to model accuracy and identifier convergence. The proposed PID augmented with a Gompertz function (PID-G) controller, in contrast, achieves its nonlinear shaping through a structurally fixed and DIO-optimized parameter set determined entirely offline, thereby avoiding the need for online adaptation while still accommodating the nonlinear characteristics of the electro-hydraulic actuator through the parametric flexibility of the Gompertz term. These distinctions indicate that the contribution of the present study, while positioned within the broader nonlinear PID framework, is not merely an incremental substitution of one nonlinear element for another, but rather a deliberate exploitation of the Gompertz function’s unique asymmetric and multi-parametric properties for transient shaping in electro-hydraulic displacement control.

**Figure 5:**
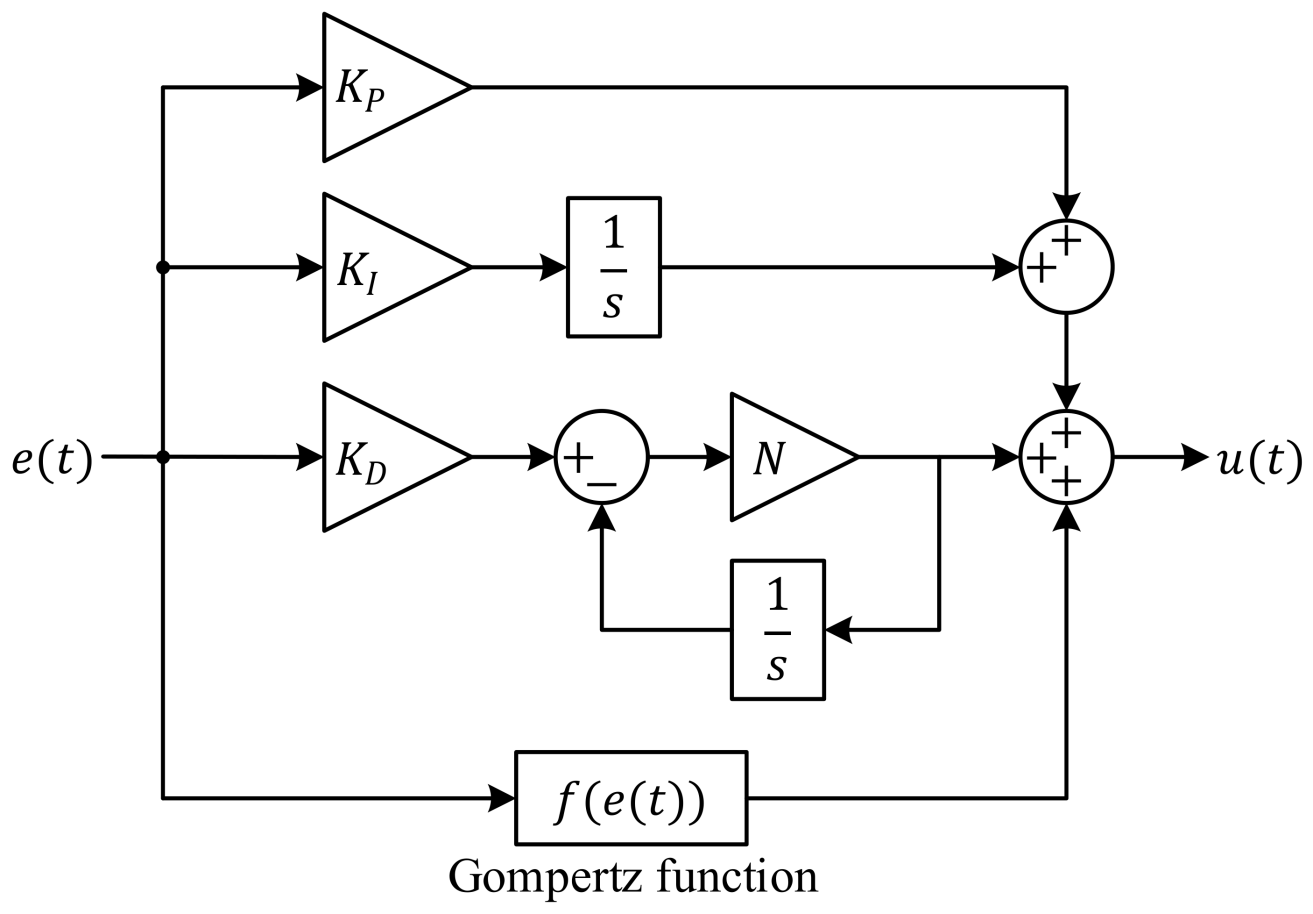
Block diagram of proposed PID-G controller.

The structure of the proposed PID augmented with a Gompertz function (PID-G) controller is shown in Figure 5. As can be observed, the tracking error e(t) is processed simultaneously through the proportional, integral, and derivative channels of the PID controller. In addition, a nonlinear branch based on the Gompertz function is incorporated in parallel with the classical PID terms. The derivative path also includes a first-order filtering action through the coefficient N, which is introduced to reduce the adverse effect of high-frequency amplification and to obtain a more practically realizable derivative component. Consequently, the resulting controller preserves the basic advantages of PID control while extending its flexibility through a nonlinear error-dependent action. According to the block structure given in Figure 5, the total output of the proposed PID-G controller is expressed in the time domain as

(21) $$u(t)=K_{P}\;e(t)+K_{I}\int_{0}^{t}e(\tau )\;d\tau +d_{f}(t)+f(e(t))$$

where $e(t)=z_{\mathrm{ref}}(t)-z_{\mathrm{out}}(t)$ denotes the tracking error, $K_{P}$, $K_{I}$, and $K_{D}$ are the proportional, integral, and derivative gains, respectively, $d_{f}(t)$ is the filtered derivative signal, and f(e(t)) is the nonlinear Gompertz contribution. The four terms represent the proportional, integral, filtered derivative, and nonlinear branches visible in the block diagram of Figure 5, each of which acts in parallel on the instantaneous tracking error.

The filtered derivative component $d_{f}(t)$ is implemented through a first-order filter applied to the derivative channel in order to attenuate the amplification of high-frequency noise that would otherwise occur with a pure differentiator. In the Laplace domain, this component is expressed as

(22) $$\frac{D_{f}(s)}{E(s)}=\frac{K_{D}Ns}{s+N}$$

where N is the filter coefficient that governs the bandwidth of the derivative action. A larger value of N brings the filtered derivative closer to an ideal differentiator, whereas a smaller value provides stronger attenuation of high-frequency components. By incorporating this filter, the derivative branch retains its transient-improvement capability while remaining practically realizable. The nonlinear term f(e(t)), embedded in the fourth branch of the controller, is defined as

(23) $$f\left(e(t)\right)=a\times exp(-b\times exp(-c\times e(t)))$$

where e(t) denotes the tracking error, and a, b, and c are the adjustable parameters of the Gompertz function. This expression indicates that the nonlinear contribution is generated directly from the instantaneous error rather than from time. Therefore, the controller output is modified in accordance with the current tracking condition. For relatively small errors, the nonlinear term changes smoothly, which is beneficial for avoiding excessive oscillatory behavior near the setpoint. For larger errors, the same term can provide an enhanced corrective influence depending on the selected values of a, b, and c. In this way, the proposed PID-G structure offers an additional degree of freedom beyond conventional PID tuning. For fixed a,b,c>0 and for arbitrary e(t)∈R (encompassing both positive tracking errors and the negative errors that accompany overshoot) the term defined in Equation (23) satisfies: (i) 0<f(e(t))<a for every finite e(t); (ii) f is strictly increasing in e(t), since $df/de\left(t\right)=abce^{-ce(t)}e^{-be^{-ce\left(t\right)}}>0$ for all e(t)∈R; (iii) ${lim}_{e(t)\to +\infty }f(e(t))=a$ and ${lim}_{e(t)\to -\infty }f(e(t))=0$, so the bounds 0 and a are approached asymptotically but never attained. Since c>0, the term $e^{-ce(t)}$ is strictly positive for every finite e(t), whether the tracking error is positive or negative. Because b>0, it follows that $-be^{-ce(t)}<0$ for all e(t), and since the exponential of any negative real number lies strictly in the open interval (0,1), $e^{-be^{-ce(t)}}\in (0,1)$ for every e(t)∈R. Multiplying by a>0 gives $f(e(t))=a\;e^{-be^{-ce(t)}}\in (0,a)$ for all e(t), which proves (i) independently of the sign of the error. Differentiating f with respect to e(t) yields Equation (23); as a, b, c, $e^{-ce(t)}$, and $e^{-be^{-ce(t)}}$ are all strictly positive for every finite e(t), the derivative is strictly positive everywhere, which proves (ii). Property (iii) follows from the limiting behavior of $e^{-ce(t)}$: as e(t)→+∞, $e^{-ce(t)}\to 0^{+}$, so $f(e(t))\to a\;e^{0}=a$; as e(t)→−∞, $e^{-ce(t)}\to +\infty$, so the inner exponent $-be^{-ce(t)}\to -\infty$ and f(e(t))→a⋅0=0. An important consequence of this result concerns the overshoot condition raised above: since a negative error increases $e^{-ce(t)}$ rather than decreasing it, negative excursions of e(t) drive f(e(t)) toward its lower bound of zero rather than amplifying the nonlinear contribution. The Gompertz branch therefore saturates, rather than grows, under overshoot, and the bound 0<f(e(t))<a holds unconditionally across the entire operating range of the tracking error, for any admissible choice of positive a, b, and c, without requiring any sign-dependent handling beyond the positivity already imposed on the shape parameters.

From a control perspective, the proposed strategy combines the well-established regulation capability of the PID mechanism with the shaping ability of the Gompertz nonlinearity. The proportional term contributes to the immediate correction of the error, the integral term improves steady-state accuracy, and the derivative term supports damping and transient improvement. The added Gompertz branch supplements these actions by introducing a bounded and continuously varying nonlinear component. Such a structure is particularly suitable for electro-hydraulic systems, whose dynamics are often affected by nonlinear flow-pressure relationships and strong transient variations. Accordingly, the PID-G controller is expected to provide improved transient shaping and tracking performance compared with conventional linear PID-based alternatives. Since the proposed controller contains seven adjustable parameters, namely $K_{P}$, $K_{I}$, $K_{D}$, N, a, b, and c, an effective tuning procedure is required in order to obtain a suitable trade-off among rise time, settling time, overshoot, and steady-state accuracy. For this reason, the controller parameter selection is formulated as an optimization problem in the next section, where the DIO is employed to determine the optimal parameter set for the electro-hydraulic actuator displacement control problem.

## 5. Application of DIO and Definition of Optimization Problem

Since the proposed PID-G structure contains seven adjustable parameters, namely $K_{P}$, $K_{I}$, $K_{D}$, N, a, b, and c, the controller design problem was formulated as a constrained optimization task and solved by means of the DIO. In this framework, each candidate solution generated by DIO represents a complete parameter set of the PID-G controller, and its quality is evaluated according to the closed-loop displacement response of the electro-hydraulic actuator. In this way, the search process is directed toward parameter combinations that provide a desirable compromise among rapid response, low overshoot, and high steady-state accuracy. To tune the PID-G controller in a performance-oriented manner, the following objective function was employed:

(24) $$OF=\left(\frac{1-e^{-\varphi }}{100}\right)\left(OS+e_{ss}\right)+e^{-\varphi }(t_{s}-t_{r})$$

where φ is the balancing coefficient and set to φ=0.5, OS is the overshoot (%), $e_{ss}$ is the steady-state error (%) evaluated at t=1 s, $t_{s}$ is the settling time defined with respect to the ±2% tolerance band, and $t_{r}$ is the rise time. The structure of Equation (24) shows that the optimization problem was not defined on the basis of a single transient indicator. Instead, a weighted performance index was established so that the main dynamic characteristics of the actuator response could be considered simultaneously. The first term penalizes excessive overshoot and residual steady-state error, whereas the second term reflects the transient speed through the difference between settling time and rise time. Accordingly, lower values of OF correspond to responses that rise rapidly, settle in a short time, and remain close to the reference with minimal overshoot and negligible steady-state deviation. This formulation was therefore adopted to ensure that the selected controller parameters would not favor only fast motion or only accuracy, but a balanced closed-loop behavior.

The search intervals of the PID-G controller parameters were selected as $0.01\leq K_{P}\leq 0.02$, $0.0001\leq K_{I}\leq 0.0008$, $0.0001\leq K_{D}\leq 0.0005$, 0.5≤N≤5, 0.1≤a≤0.5, 1≤b≤10, and 1≤c≤20. These bounds define the feasible search region explored by DIO during the optimization process. By constraining the search in this manner, the tuning procedure was limited to practically meaningful parameter values while still preserving sufficient flexibility for the optimizer to identify an effective balance between the linear PID action and the nonlinear Gompertz contribution. The bounds adopted for the Gompertz parameters, in particular the upper limit of 10 imposed on b, were established through a preliminary trial-and-error exploration of the feasible search space rather than fixed arbitrarily. In this exploratory phase, wider intervals were also examined, including values of b beyond the reported upper limit; these trials did not yield closed-loop responses that were meaningfully superior to those obtained within the adopted bounds, since larger values of b were observed to compress the transition region of the Gompertz term without producing a discernible improvement in the objective-function value. The interval 1≤b≤10 was therefore retained as an adequate representation of the effective operating range of this parameter, and the proximity of the DIO solution to this upper limit is interpreted as evidence of the algorithm’s capability to fully exploit the defined search space rather than as an indication that the search region excluded a substantially better solution. As these preliminary checks did not point to a materially different outcome, a dedicated sensitivity analysis was not included separately in the manuscript, in order to preserve consistency with the comparative results reported in Section 6, all of which correspond to the bounds stated above.

**Figure 6:**
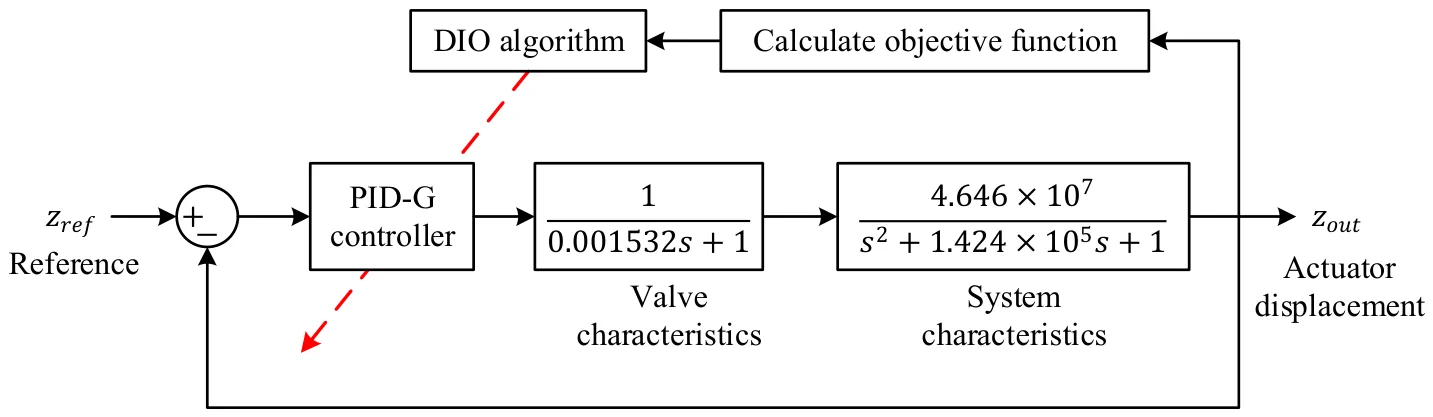
Implementation of DIO-based PID-G controller for electro-hydraulic actuator displacement.

The implementation of the optimization-based control structure is illustrated in Figure 6. As shown in the figure, the reference signal $z_{ref}$ is compared with the actuator displacement output $z_{out}$ to generate the tracking error, which is then processed by the PID-G controller. The controller output drives the valve dynamics represented by the first-order block 1/(0.001532s+1), and the resulting valve response excites the electro-hydraulic actuator dynamics described by the system block $4.646\times 10^{7}/(s^{2}+1.424\times 10^{5}s+1)$. The PID-G controller structure and its output equation are described in Section 4 (Equations (21)–(23)); the valve and actuator transfer functions are given in Section 3 (Equations (17) and (15), respectively); the numerical plant model is provided in Equation (19); and the objective function minimized by DIO during iterative parameter refinement is defined in Equation (24). On the basis of the obtained closed-loop response, the objective function is calculated and returned to the DIO, which updates the controller parameters iteratively until the best parameter set is obtained. Through this procedure, the parameter tuning of the PID-G controller was transformed into a systematic search problem in which DIO continuously refined the controller coefficients according to the time-domain performance of the electro-hydraulic actuator. The resulting optimized controller parameters and the associated comparative performance analyses are presented in the following section.

## 6. Simulation Results

In this section, the effectiveness of the proposed DIO-based tuning strategy for the PID-G controller is examined through a comprehensive set of simulation studies carried out on the four-way valve-controlled electro-hydraulic actuator model. The performance of DIO is evaluated comparatively against three well-established and high-performance optimization algorithms, namely the flood algorithm (FLA) [38], derandomized evolution strategy with covariance matrix adaptation (CMA-ES) [30] and particle swarm optimization (PSO) [39]. In this way, the relative capability of the proposed optimization framework can be assessed under identical operating and tuning conditions.

For a fair comparison, all algorithms were executed using the same experimental setup. Specifically, 30 independent runs were performed for each method, the population size was fixed at 20, and the maximum number of iterations was set to 50. Such a configuration was adopted in order to evaluate not only the best obtained solution, but also the repeatability and robustness of the optimization process under repeated stochastic trials. Since the controller design problem considered in this study involves a multi-parameter and nonlinear search space, repeated independent executions are necessary to provide a reliable basis for performance comparison. The adopted population size and iteration horizon are consistent with configurations widely used in the metaheuristic controller-tuning literature for real-world engineering problems of comparable or higher dimensionality, where a population of 20–30 candidate solutions evaluated over 50 iterations across 30 independent runs has been shown to yield reliable and repeatable tuning outcomes for multi-parameter PID-type controllers [40]. Consistent with this precedent, the convergence behavior indicates that all four compared algorithms reached a stable plateau well before the maximum iteration count was exhausted, while the low relative standard deviation obtained for DIO across the 30 independent runs (Table 2) confirms that the selected population size was sufficient to yield consistent outcomes despite the stochastic nature of the search. These observations jointly support the adequacy of the chosen hyperparameters for the seven-dimensional PID-G tuning problem considered in this study.

Beyond these shared settings, the algorithm-specific internal parameters of each optimizer (including the vocalization influence coefficient update rule of DIO, the flood propagation parameters of FLA, the covariance matrix adaptation settings of CMA-ES, and the inertia and acceleration coefficients of PSO) were set to the default values recommended in the respective original publications [20,30,38,39], without any problem-specific adjustment, in order to ensure a fair and unbiased comparative evaluation.

For completeness and reproducibility, it is noted that the electro-hydraulic actuator plant is fully defined by the numerical transfer function in Equation (19) together with the parameter values in Table 1; the PID-G controller structure is given by Equations (21)–(23); the objective function and its associated tuning bounds are provided in Equation (24) and the surrounding text of Section 5; and the best-identified controller parameter sets for all compared algorithms are listed in Table 3. All numerical data required to reproduce the deterministic components of the study (namely the plant model, the controller structure, the objective function, the parameter bounds, and the shared experimental protocol) are therefore available within the manuscript, and are sufficient for an independent implementation to replicate the experimental setup and obtain statistically consistent results. Because no fixed random seed was used across the 30 independent runs, as noted below, exact numerical reproduction of any single stochastic run is not intended by this statement; rather, the statistical outcomes summarized in Table 2 are expected to be reproducible in distribution under repeated, independently seeded trials conducted according to the same protocol. Regarding initial conditions, all closed-loop simulations were initiated from rest, with the actuator displacement, velocity, and all internal controller states set to zero at t=0, and the reference input applied as a step to the target value of 20 cm. The optimizer population was independently and randomly initialized within the stated parameter bounds at the start of each of the 30 independent runs in order to ensure an unbiased statistical assessment.

The simulation results are organized into several complementary parts. First, the minimization capability of the objective function defined in Equation (24) is examined. Then, the statistical characteristics of the obtained results are analyzed in order to assess consistency and significance. After that, the convergence behavior and the best controller parameters identified by the competing algorithms are presented. This is followed by time-domain performance comparisons, additional assessments based on different error-oriented objective functions, comparative analysis with different PID-type controller structures, frequency-domain evaluation, setpoint-tracking analysis together with controller-output behavior, and a dedicated discussion of implementation feasibility and practical considerations. Through this structure, the performance of the proposed DIO-based PID-G design is evaluated from multiple and mutually supportive perspectives.

### 6.1. Performance Evaluation of Objective Function Minimization

**Figure 7:**
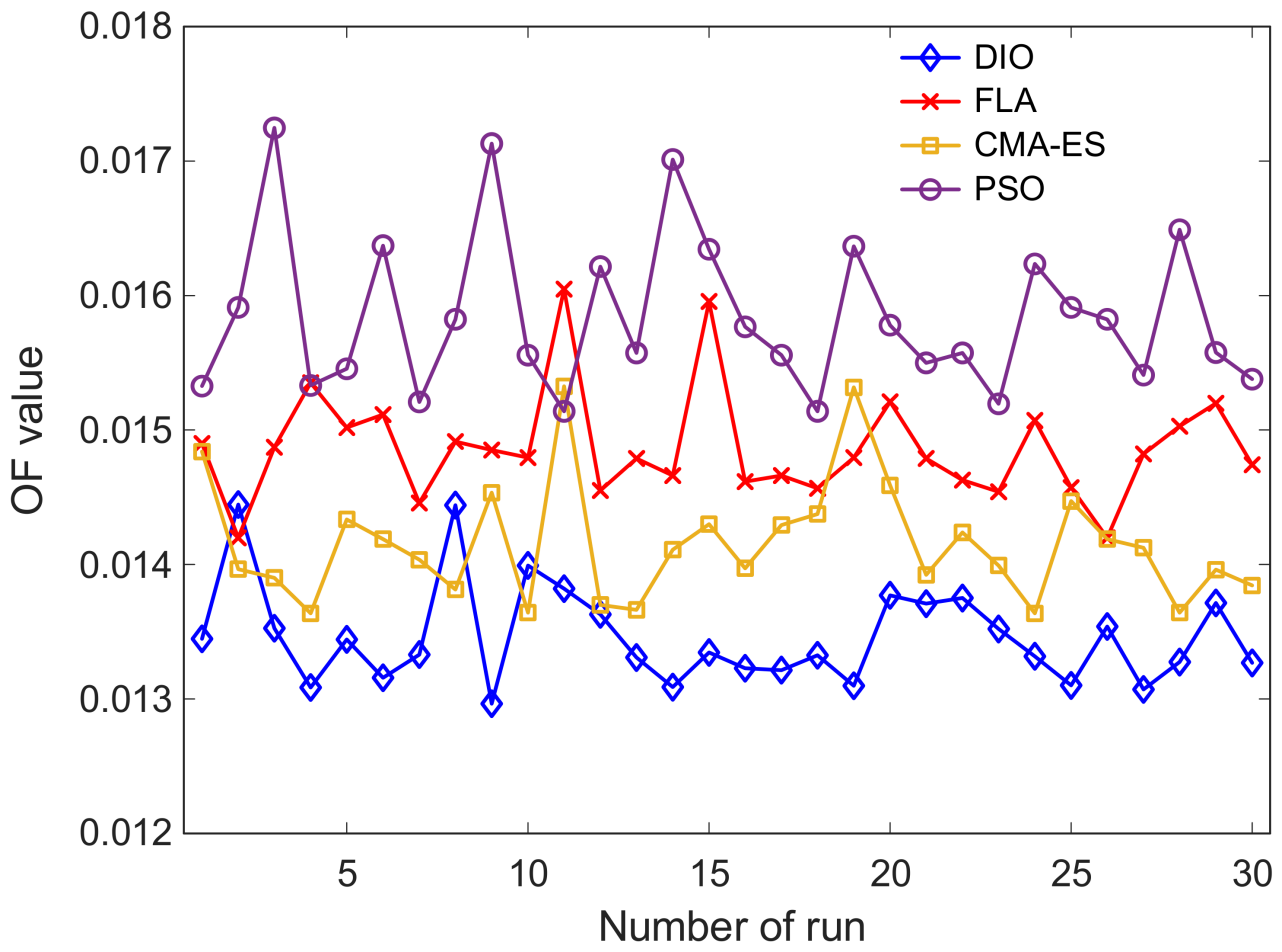
Objective function values obtained over 30 independent runs for DIO, FLA, CMA-ES, and PSO.

In order to evaluate the OF minimization capability of the considered optimization algorithms, 30 independent runs were carried out for DIO, FLA, CMA-ES, and PSO under the same population size and iteration settings. The obtained OF values for all runs are presented in Figure 7. Since the objective function defined in Equation (24) simultaneously reflects overshoot, steady-state error, rise time, and settling time, the comparative behavior shown in Figure 7 directly indicates the relative ability of each algorithm to produce controller parameters that yield a well-balanced closed-loop response. Accordingly, smaller OF values correspond to more desirable tuning results.

As can be observed from Figure 7, the DIO-based tuning procedure consistently produced the lowest objective-function values among the compared methods over the majority of the independent runs. The DIO curve remained concentrated in the lowest region of the graph, with only limited fluctuation from run to run. This behavior indicates that DIO was able not only to attain low OF values, but also to preserve a stable search performance across repeated executions. Such a result is important in the present control design problem because a reliable optimizer should provide high-quality controller parameters without exhibiting excessive sensitivity to stochastic initialization. The CMA-ES yielded the second-best overall performance in Figure 7. Its OF values generally remained below those of FLA and PSO, although they were still higher than those obtained by DIO. Moreover, its run-to-run variation was visibly larger than that of DIO, which suggests that, while CMA-ES was capable of identifying competitive solutions, its performance was not as consistently favorable. The FLA followed with intermediate performance, producing OF values that were generally higher than those of DIO and CMA-ES. Although FLA occasionally approached the performance region of CMA-ES, its results remained overall less favorable. Among the four methods, PSO exhibited the highest OF values and the most pronounced fluctuations, indicating a comparatively weaker and less stable minimization performance for the present tuning task.

From a comparative perspective, the distribution of the curves in Figure 7 shows a clear ranking in terms of OF minimization performance, with DIO providing the most favorable results, followed by CMA-ES, FLA, and PSO. This trend suggests that the search dynamics of DIO were more effective in identifying parameter combinations of the PID-G controller that better satisfied the multi-criteria performance objective formulated in Equation (24). Therefore, the results of Figure 7 provide an initial indication that DIO offers a more robust and effective optimization capability for the electro-hydraulic actuator displacement-control problem considered in this study.

### 6.2. Statistical Performance Evaluation

**Figure 8:**
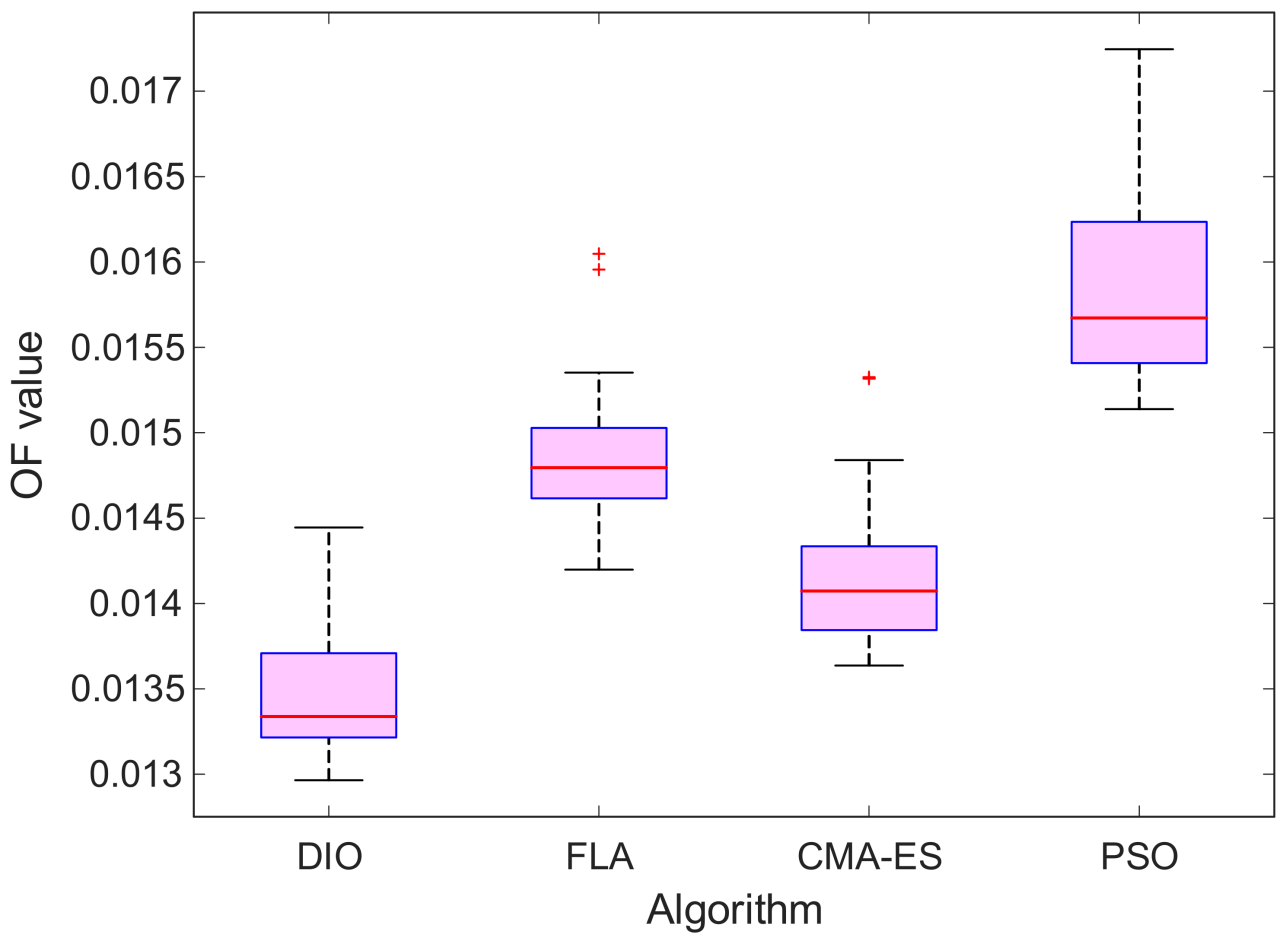
Boxplot representation of the objective function values obtained over 30 independent runs for DIO, FLA, CMA-ES, and PSO.

In addition to the run-based comparison presented in Figure 7, the statistical characteristics of the obtained objective-function values were examined in order to assess the consistency, dispersion, and relative reliability of the competing algorithms over 30 independent runs. For this purpose, the distribution of the results is illustrated by the boxplot in Figure 8, while the corresponding best, worst, average, standard deviation, and Wilcoxon test results are summarized in Table 2. As shown in Figure 8, the DIO results are concentrated in the lowest region among all compared algorithms. The median value of DIO is clearly lower than those of FLA, CMA-ES, and PSO, and its interquartile range is relatively narrow. This distribution indicates that DIO not only achieved lower objective-function values, but also maintained a comparatively stable performance under repeated executions. In contrast, the distributions of FLA and PSO are shifted toward higher OF levels, while CMA-ES occupies an intermediate position between DIO and the remaining methods. The broader spread observed especially for PSO suggests greater variability in the obtained solutions, which reflects a less consistent optimization behavior for the present controller tuning problem.

The numerical findings reported in Table 2 support the visual interpretation of Figure 8. DIO yielded the best overall statistical performance with a best value of 0.012965, a worst value of 0.014446, and an average value of 0.013465. These results were lower than the corresponding values obtained by the other algorithms. CMA-ES followed as the second-best method, with best, worst, and average values of 0.013637, 0.015325, and 0.014152, respectively. FLA produced slightly higher values, with a best value of 0.014199 and an average value of 0.014864. PSO exhibited the least favorable statistical performance, with the highest average value of 0.015845 and the highest worst-case value of 0.017246. Therefore, the ranking inferred from the raw run data remains unchanged when the results are evaluated statistically.

A similar tendency is observed in the standard deviation values. DIO produced the lowest standard deviation, namely 0.00037145, which indicates the smallest run-to-run variation among the compared approaches. FLA and CMA-ES yielded slightly higher standard deviations of 0.00041039 and 0.00044505, respectively, whereas PSO showed the largest dispersion with a standard deviation of 0.00058489. These results confirm that DIO provided not only the lowest objective-function values on average, but also the most repeatable optimization performance. In the context of controller tuning, such repeatability is particularly important because it indicates that the optimizer is less sensitive to stochastic initial conditions and is more likely to produce reliable parameter sets in repeated trials.

The nonparametric Wilcoxon test results listed in Table 2 further reinforce this conclusion. The *p*-values obtained for the comparisons against DIO are $1.9209\times 10^{-6}$ for FLA, $2.8434\times 10^{-5}$ for CMA-ES, and $1.7344\times 10^{-6}$ for PSO. These very small values indicate that the observed differences between DIO and the competing algorithms are statistically significant. Hence, the superiority of DIO is not limited to isolated runs or visual trends alone, but is also supported by statistical evidence derived from repeated experiments.

### 6.3. Evolution of the Objective Function and the Obtained Best Controller Parameters

**Figure 9:**
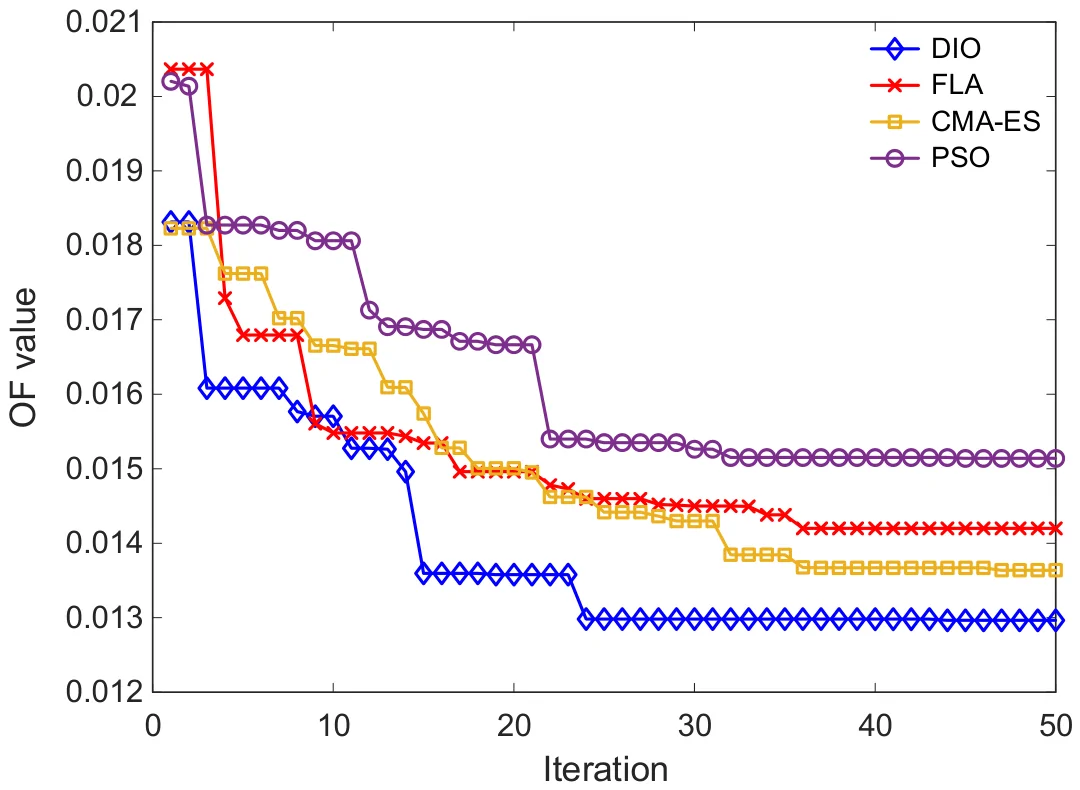
Convergence curves of the objective function over 50 iterations for DIO, FLA, CMA-ES, and PSO.

The convergence characteristics of the compared optimization algorithms are presented in Figure 9, and the best parameter sets obtained for the PID-G controller are listed in Table 3. This part of the analysis is important because the quality of an optimization method in controller tuning is determined not only by the final objective-function value, but also by the manner in which the solution is approached during the iterative search process. In this regard, Figure 9 provides direct insight into the exploration–exploitation behavior of DIO, FLA, CMA-ES, and PSO, whereas Table 3 identifies the corresponding controller parameters associated with the best solutions. As shown in Figure 9, all four algorithms reduced the objective-function value as the number of iterations increased, which indicates that each method was capable of improving the controller parameters progressively throughout the search process. Nevertheless, clear differences are observed in both convergence speed and final convergence level. The DIO curve descended rapidly during the early iterations and continued to improve until it reached the lowest final level among all compared methods. This behavior suggests that DIO was able to exploit promising regions of the search space effectively after an initial exploration stage, thereby achieving a better balance between search diversification and refinement. The convergence profile of CMA-ES can be regarded as the second most favorable. Although its objective-function value decreased steadily and reached a competitive final level, the terminal value remained above that of DIO. FLA also showed continuous improvement throughout the optimization process; however, its convergence trajectory remained consistently higher than those of DIO and CMA-ES. Among the compared methods, PSO exhibited the slowest and least favorable convergence pattern. Its curve decreased more gradually and stabilized at the highest final objective-function level, indicating that its search process was less effective in locating highly favorable PID-G parameter combinations for the present problem.

From a comparative standpoint, Figure 9 confirms that DIO provided both a faster convergence tendency and a lower terminal objective-function value than the alternative algorithms. This finding is consistent with the run-based and statistical analyses presented in the preceding subsections. More specifically, the lower asymptotic level reached by DIO indicates that the algorithm was able to guide the search toward a more desirable compromise among overshoot, steady-state error, rise time, and settling time, all of which are embedded in the objective function defined in Equation (24). Therefore, the superiority of DIO is reflected not only in the final numerical results, but also in the evolution of the search process itself.

The best controller parameters obtained by the compared algorithms are summarized in Table 3. According to these results, the DIO-based PID-G controller was obtained with $K_{P}=0.018219$, $K_{I}=0.00048590$, $K_{D}=0.00035709$, N=0.84914, a=0.43762, b=9.6039, and c=13.2739. The corresponding parameter sets identified by FLA, CMA-ES, and PSO differ noticeably, which indicates that each optimizer converged to a different region of the feasible search space. This variation is expected because the controller includes both linear PID gains and nonlinear Gompertz parameters, and the interaction among these seven variables yields a multi-parameter optimization landscape with multiple competing trade-offs.

A closer inspection of Table 3 shows that the obtained parameter values are not merely minor perturbations of one another, but represent distinct tuning configurations. For example, the DIO solution is associated with comparatively high values of $K_{P}$, b, and c, while preserving intermediate values of $K_{I}$, $K_{D}$, and a. In contrast, CMA-ES yields lower values of N and different Gompertz parameters, whereas PSO produces a notably smaller value of a together with a lower value of b. These differences indicate that the improvement achieved by DIO cannot be attributed to a trivial numerical coincidence. Rather, it reflects the ability of the algorithm to locate a parameter combination that more effectively coordinates the linear feedback terms and the nonlinear Gompertz contribution within the PID-G controller structure.

### 6.4. Time-Domain Performance Evaluation

**Figure 10:**
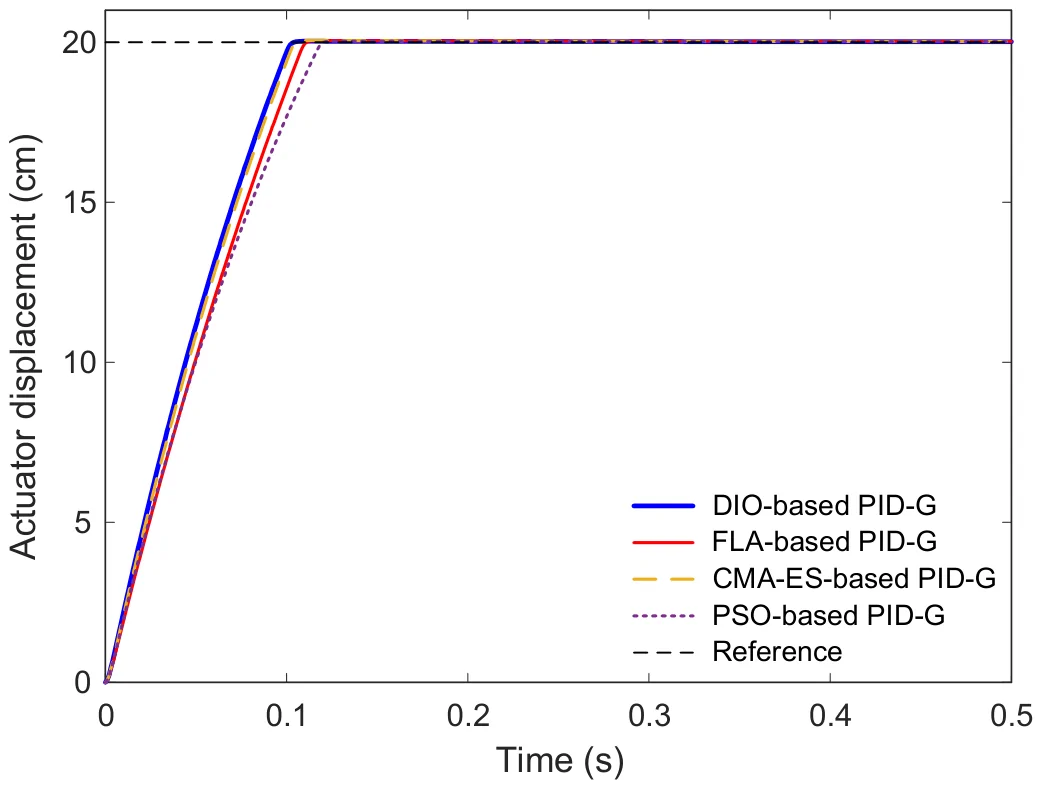
Time-domain responses of the DIO-, FLA-, CMA-ES-, and PSO-based PID-G controllers for electro-hydraulic actuator displacement tracking.

The time-domain responses obtained with the PID-G controllers tuned by DIO, FLA, CMA-ES, and PSO are presented in Figure 10, and the corresponding numerical performance indices are summarized in Table 4. Since the objective function was formulated by considering overshoot, steady-state error, rise time, and settling time simultaneously, the time-domain analysis provides the most direct verification of whether the optimization results translate into practically desirable closed-loop behavior. In this respect, the response curves and the associated transient indices allow the comparative effectiveness of the tuned controllers to be assessed in terms of tracking speed, overshoot suppression, and steady-state accuracy. As shown in Figure 10, all optimized PID-G controllers were able to drive the actuator displacement to the 20 cm reference value with a stable and nonoscillatory response. Nevertheless, noticeable differences are observed in the transient portion of the responses. The DIO-based PID-G controller reached the reference more rapidly than the competing methods and exhibited the most favorable overall transient profile. Its response rose quickly, settled earlier, and remained very close to the reference trajectory with only a very small peak excursion. The CMA-ES-based controller followed with a response that was also fast, but slightly less favorable than that of DIO. The FLA-based controller exhibited a slower rise and settling process than DIO and CMA-ES. Among the compared methods, the PSO-based controller showed the slowest approach to the reference, which is consistent with the less favorable optimization performance observed in the earlier subsections.

The numerical results reported in Table 4 confirm the visual trends seen in Figure 10. The DIO-based PID-G controller achieved the lowest rise time, $t_{r}=0.079511$ s, and the lowest settling time, $t_{s}=0.099326$ s, among all four approaches. In addition, it produced a maximum peak of $M_{p}=20.030$ cm and an overshoot of only OS=0.15110%, together with a peak time of $t_{p}=0.1120$ s and a steady-state error of $e_{ss}=0.089317\%$. These values indicate that the DIO-based design provided the fastest response while preserving very good tracking precision and limited overshoot. Such a combination is particularly desirable for electro-hydraulic positioning systems, where both response speed and accuracy are important performance requirements. The CMA-ES-based PID-G controller can be regarded as the second-best alternative in terms of rise time and settling time, with $t_{r}=0.081246$ s and $t_{s}=0.10135$ s, respectively. However, its overshoot and maximum peak values, OS=0.27395% and $M_{p}=20.055$ cm, were higher than those of DIO. The FLA-based controller yielded $t_{r}=0.085974$ s and $t_{s}=0.10699$ s, together with an overshoot of 0.24428% and a steady-state error of 0.12499%, indicating a weaker overall transient performance than both DIO and CMA-ES. The PSO-based controller exhibited the largest rise time and settling time, $t_{r}=0.092410$ s and $t_{s}=0.11553$ s, respectively, although its overshoot remained lower than those of FLA and CMA-ES. Even so, its steady-state error of 0.11749% and slower transient response indicate a less favorable overall balance compared with DIO.

It is worth clarifying why a small but nonzero steady-state error is reported for all four controllers in Table 4 despite the presence of integral action. The plant model of Equation (19) is not itself a Type 1 system: its denominator retains a nonzero constant term, so the corresponding transfer function has no pole at the origin, and the sole integrator in the closed loop originates from the controller’s own $K_{I}$ term rather than from the plant. Nevertheless, because the constant term in the denominator of Equation (19) is small relative to the remaining coefficients, the plant possesses a very slow, near-integrating mode whose associated time constant is several orders of magnitude longer than the transient interval captured by $t_{r}$ and $t_{s}$. As specified in Section 5, $e_{ss}$ is evaluated at t=1 s rather than in the infinite-horizon limit, and this evaluation instant, although appropriate for capturing the practically relevant transient behavior of the actuator, occurs well before this slow residual mode has fully decayed. The nonzero values reported in Table 4 therefore reflect this finite-time evaluation rather than a persistent tracking offset, and are consistent with the theoretical property that integral action drives the steady-state error asymptotically to zero. This effect is common to all four compared algorithms, since they share the same plant dynamics, and is unrelated to the Gompertz branch, whose contribution decays to a negligible level well within the rise-time interval given the steepness parameters identified in Table 3, and is likewise unrelated to numerical integration tolerance, since it arises from the intrinsic separation of time scales in the plant model rather than from the discretization of the simulation.

When the time-domain indices are considered jointly, the DIO-based PID-G controller clearly offers the best compromise among rapid rise, short settling time, low overshoot, and small steady-state error. This result is fully consistent with the previous findings obtained from the objective-function, statistical, and convergence analyses. In other words, the numerical advantage of DIO in the optimization stage is not merely algorithmic in nature; it also leads directly to improved closed-loop control performance in the time domain. From a practical viewpoint, the results in Figure 10 and Table 4 indicate that the DIO-optimized controller is more capable of providing fast and accurate actuator positioning than the alternative optimization-based PID-G designs considered in this study. The very small overshoot values obtained for all methods already demonstrate that the controller structure is effective in limiting excessive transient excursion. However, the DIO-based solution distinguishes itself by reaching the desired displacement faster and with lower residual error. Therefore, the time-domain analysis confirms that the DIO-based PID-G controller constitutes the most effective tuning outcome among the compared methods for the electro-hydraulic actuator displacement-control problem addressed in this work.

### 6.5. Performance Evaluation with Respect to Different Error Based Objective Functions

In addition to the composite objective function defined in Equation (24), the closed-loop responses obtained with the compared optimization algorithms were further examined by means of widely used error-based integral performance criteria. For this purpose, the integral absolute error (IAE), integral squared error (ISE), integral time absolute error (ITAE), and integral time squared error (ITSE) were considered, as given in Equations (25)–(28), with the final evaluation time selected as $t_{f}=1$ s. These indices provide complementary information on the tracking quality of the actuator response, because they quantify the accumulated error from different perspectives. While IAE and ISE reflect the overall magnitude of the tracking error, ITAE and ITSE impose additional time weighting and therefore penalize errors that persist for a longer duration more severely. Accordingly, lower values of these indices indicate a more desirable control performance:

(25) $$IAE=\int_{0}^{t_{f}}\left|e(t)\right|dt$$

(26) $$ISE=\int_{0}^{t_{f}}e^{2}(t)dt$$

(27) $$ITAE=\int_{0}^{t_{f}}t\left|e(t)\right|dt$$

(28) $$ITSE=\int_{0}^{t_{f}}te^{2}(t)dt$$

The numerical results corresponding to these four criteria are summarized in Table 5. As can be observed, the DIO-based PID-G controller achieved the lowest value in all four metrics, namely IAE=0.95041 cm-s, ISE=12.053 cm^2^-s, ITAE=0.038689 cm-s^2^, and ITSE=0.27922 cm^2^-s^2^. These results indicate that the controller parameters obtained by DIO yielded the smallest accumulated tracking error over the evaluation interval, irrespective of whether the error was assessed in absolute, squared, or time-weighted form. Hence, the superiority of DIO is not restricted to the specific composite objective function adopted for tuning, but is also maintained when the response quality is examined through alternative and widely accepted error measures. Among the remaining methods, CMA-ES provided the second-best overall performance across all four criteria. Its results, IAE=0.98261 cm-s, ISE=12.434 cm^2^-s, ITAE=0.042261 cm-s^2^, and ITSE=0.29622 cm^2^-s^2^, were consistently lower than those of FLA and PSO, although still higher than those of DIO. The FLA-based solution followed with IAE=1.0522 cm-s, ISE=13.395 cm^2^-s, ITAE=0.048435 cm-s^2^, and ITSE=0.34172 cm^2^-s^2^. The PSO-based controller produced the largest values for all four metrics, namely IAE=1.0828 cm-s, ISE=13.555 cm^2^-s, ITAE=0.050537 cm-s^2^, and ITSE=0.35838 cm^2^-s^2^, indicating the least favorable overall tracking accuracy among the compared methods.

A noteworthy point is that the ranking observed in Table 5 is fully consistent with the trends previously identified through the objective-function, statistical, convergence, and time-domain analyses. In all cases, DIO remains the best-performing method, followed by CMA-ES, FLA, and PSO. This consistency is important because it shows that the superiority of the DIO-based tuning strategy is not an artifact of a single performance definition. On the contrary, the DIO-optimized controller continues to exhibit the most favorable behavior even when evaluated under different error criteria that emphasize different aspects of the transient response. From a control perspective, the lower IAE and ISE values obtained by DIO indicate that the total magnitude of the tracking error and the energy-like accumulation of the error were reduced more effectively than with the other methods. Similarly, the lower ITAE and ITSE values demonstrate that DIO was particularly successful in suppressing errors that persist over time, which is directly associated with faster settling and more effective error elimination during the later stages of the response. Therefore, the results of Table 5 provide additional evidence that the DIO-based PID-G controller offers the most favorable balance between transient rapidity and sustained tracking accuracy for the electro-hydraulic actuator displacement-control problem considered in this study.

### 6.6. Performance Evaluation with Respect to Different Variants of the Pid Controller

In order to further assess the effectiveness of the proposed PID-G structure, an additional comparative study was carried out against several established PID-based controller variants tuned by the same DIO. For this purpose, proportional-integral (PI) [41], proportional-integral-derivative (PID) [42], two-degree-of-freedom PID (2DOF-PID) [43], and fractional-order PID (FOPID) [44] controllers were considered, as given in Equations (29)–(32). In this way, the contribution of the Gompertz-based nonlinear augmentation can be evaluated more clearly under a common optimization framework. Since all controller types were tuned by the same optimizer, the differences observed in the results can be attributed primarily to the controller structures themselves rather than to variations in the optimization method. The considered controller forms are expressed as follows:

(29) $$U\left(s\right)=\left(K_{P}+\frac{K_{I}}{s}\right)E(s)$$

(30) $$U\left(s\right)=\left(K_{P}+\frac{K_{I}}{s}+K_{D}\frac{Ns}{s+N}\right)E(s)$$

(31) $$U\left(s\right)=K_{P}\left[\beta R\left(s\right)-Y(s)\right]+\frac{K_{I}}{s}E(s)+K_{D}\frac{Ns}{s+N}\left[\gamma R\left(s\right)-Y(s)\right]$$

(32) $$U(s)=\left(K_{P}+\frac{K_{I}}{s^{\lambda }}+K_{D}s^{\mu }\right)E(s)$$

The DIO-optimized parameter values obtained for these controllers are listed in Table 6. As expected, the parameter sets differ according to the structural flexibility of each controller. The PI controller requires only $K_{P}$ and $K_{I}$, whereas the PID and 2DOF-PID structures incorporate derivative-related parameters, and the FOPID controller introduces the additional fractional orders λ and μ. The proposed PID-G controller, on the other hand, extends the conventional PID framework by combining filtered derivative action with the nonlinear Gompertz parameters. Consequently, it provides a broader shaping capability than the purely linear alternatives.

**Figure 11:**
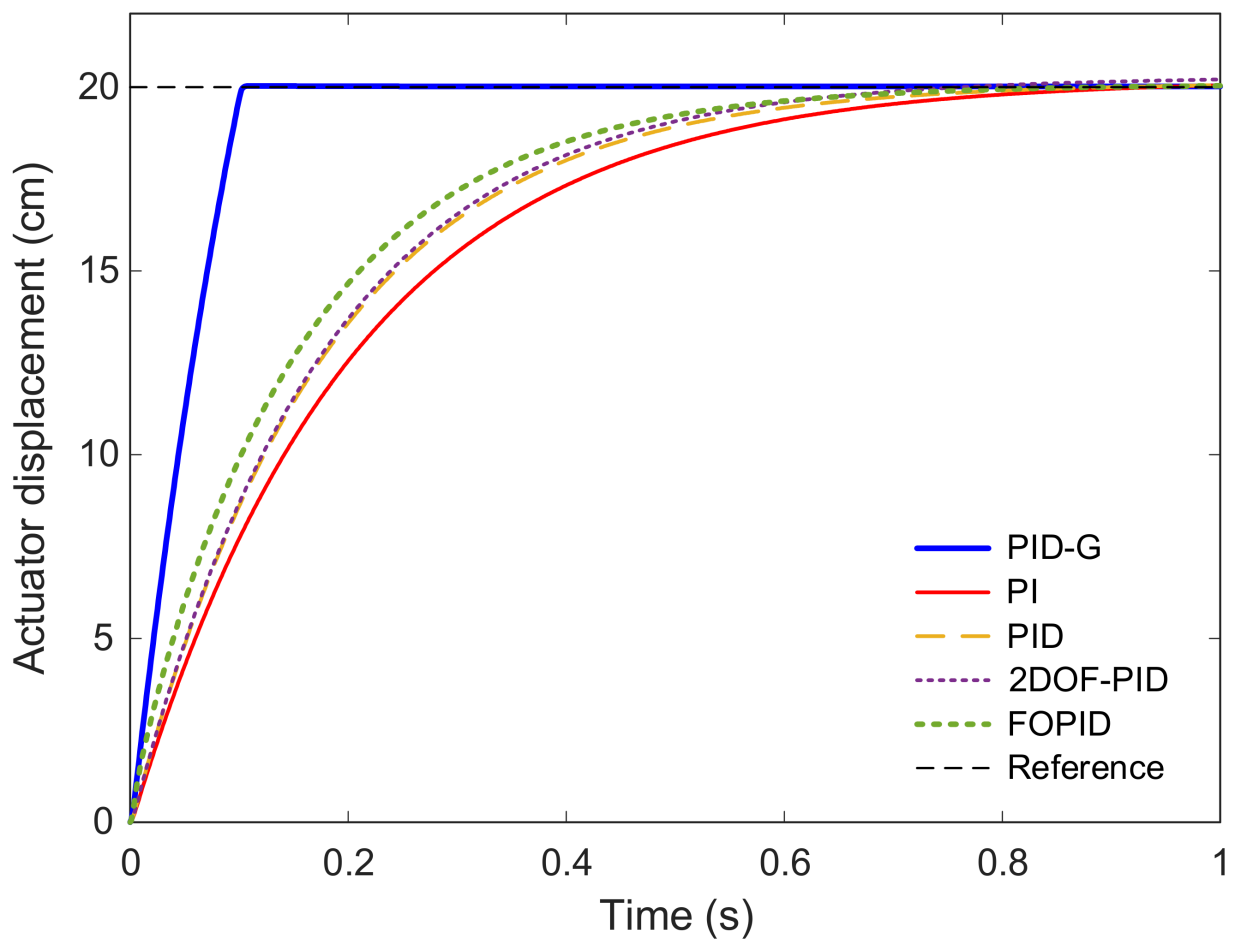
Comparative time-domain responses of the DIO-optimized PID-G, PI, PID, 2DOF-PID, and FOPID controllers for electro-hydraulic actuator displacement tracking.

The comparative time responses of the different controllers are presented in Figure 11. It can be seen that all controller structures were able to track the 20 cm reference without instability. However, the transient profiles differ markedly. The PID-G controller exhibits the fastest rise and the earliest arrival to the reference level, and its response settles very quickly with only a very small peak excursion. In contrast, the PI, PID, 2DOF-PID, and FOPID responses rise much more slowly and require a substantially longer interval to approach the reference value. This visual trend already indicates that the nonlinear augmentation introduced through the Gompertz term improves the transient shaping capability of the controller beyond what is achieved by the considered linear and fractional-order alternatives.

The numerical results in Table 7 confirm this observation. The PID-G controller achieved the best overall time-domain performance with $t_{r}=0.079511$ s, $t_{s}=0.099326$ s, $M_{p}=20.030$ cm, OS=0.15110%, $t_{p}=0.1120$ s, and $e_{ss}=0.089317\%$. These values are substantially better than those obtained by the remaining controllers. The PI controller exhibited the slowest response, with $t_{r}=0.43161$ s and $t_{s}=0.71933$ s. The classical PID controller improved upon PI, yet its rise and settling times, 0.37916 s and 0.64803 s, remained far above those of PID-G. The 2DOF-PID controller produced a somewhat faster response than PI and PID, but it resulted in the largest overshoot, 1.0227%, and the highest maximum peak, 20.205 cm. The FOPID controller yielded the best performance among the alternative benchmark controllers, with $t_{r}=0.34096$ s and $t_{s}=0.59271$ s, and also maintained a relatively low overshoot of 0.16685%. Even so, its performance remained clearly inferior to that of PID-G in both speed and overall accuracy.

A similar conclusion is obtained from the error-based integral criteria summarized in Table 8. The PID-G controller again yielded the lowest values for all metrics, namely IAE=0.95041 cm-s, ISE=12.053 cm^2^-s, ITAE=0.038689 cm-s^2^, and ITSE=0.27922 cm^2^-s^2^. These results show that the accumulated tracking error over the evaluation interval was minimized most effectively by the proposed controller. In comparison, the PI controller produced the largest error indices, with IAE=3.9298 cm-s and ITSE=4.0169 cm^2^-s^2^, indicating the weakest overall tracking quality. The PID and 2DOF-PID controllers improved upon PI, whereas the FOPID controller delivered the most favorable results among the alternatives, with IAE=2.9873 cm-s, ISE=29.241 cm^2^-s, ITAE=0.44770 cm-s^2^, and ITSE=2.2518 cm^2^-s^2^. Nevertheless, all of these values remain considerably higher than those of PID-G. Taken together, the results of Figure 11 and Table 7 and Table 8 demonstrate that the proposed PID-G controller offers a substantial improvement over the considered PI, PID, 2DOF-PID, and FOPID structures when all are optimized under the same DIO framework. This improvement is reflected not only in faster rise and settling characteristics, but also in lower overshoot, smaller steady-state error, and reduced integrated tracking error. Therefore, the benefit of the proposed controller does not arise solely from parameter tuning, but from the additional nonlinear shaping capability introduced by the Gompertz function. For the electro-hydraulic actuator displacement-control problem considered in this study, this added flexibility enables a more effective balance between response rapidity and tracking precision than that achieved by the benchmark controller variants.

The magnitude of this improvement can be explained directly from the structural properties of the Gompertz term established in Section 4, taken together with the DIO-optimized parameters listed in Table 3. Because the closed-loop simulations are initiated from rest against the 20 cm step reference specified in Section 6, the tracking error is large and positive throughout the early part of the transient phase. Given the steepness parameter identified by DIO, c=13.2739, the exponent −c e(t) in Equation (20) is strongly negative under such conditions, so the inner exponential term collapses to a value indistinguishable from zero and f(e(t)) sits, from the outset of the response, at a level effectively equal to its upper bound, a=0.43762, consistent with the limiting behavior proven in Section 4. Unlike the proportional, integral, and filtered-derivative terms, whose magnitudes are governed by the comparatively modest linear gains obtained by DIO, the Gompertz branch is therefore able to supply a substantial, near-saturated corrective contribution throughout the large-error phase of the transient, independently of these gains. Because none of the benchmark controllers considered in this study possesses a comparable structural element, their transient speed remains constrained by the magnitude of their linear or fractional-order gains alone, which accounts for the considerably faster rise achieved by PID-G in Table 7. At the same time, the strict monotonicity of f(e(t)) in e(t), also established in Section 4, ensures that this contribution recedes toward its lower bound as the tracking error diminishes near the reference, so that the additional corrective action is withdrawn automatically as the setpoint is approached rather than persisting into the steady-state region. This behavior is consistent with the low overshoot value reported for PID-G in Table 7 and confirms that the improvement in rise time is a direct and analytically grounded consequence of the Gompertz term’s saturating, monotonic character, rather than an unexplained numerical outcome of the optimization process.

### 6.7. Frequency-Domain Performance Analysis

In order to complement the time-domain evaluations presented in the preceding subsections, the frequency-domain characteristics of the DIO-optimized PID-G controller were also examined by means of the linearized open-loop response. This analysis is useful because it provides additional insight into the stability margin and dynamic robustness of the proposed control structure from a frequency-response perspective, and it allows certain theoretical stability properties of the closed-loop system to be assessed in an analytically tractable manner. Before presenting the numerical findings, it is worthwhile to discuss the theoretical stability implications of the proposed controller structure. The Gompertz nonlinear term embedded in the PID-G controller is defined as f(e(t))=a⋅exp(−b⋅exp(−c⋅e(t))). As established formally in Section 4, the function is globally bounded and strictly monotonic for all e(t)∈R, satisfying 0<f(e(t))<a regardless of the magnitude or sign of the tracking error, with negative excursions of e(t) driving the term toward its lower bound rather than amplifying it. This uniform boundedness is significant from a stability perspective because it ensures that the nonlinear branch of the controller cannot generate an unbounded control contribution under any error condition, thereby precluding the possibility of finite-escape-time instability arising from the Gompertz term alone. Consequently, the nonlinear augmentation can be treated analytically as a bounded additive perturbation superimposed on the linear PID loop, and the stability of the overall closed-loop system can be assessed, to a first approximation, by examining the linearized feedback structure.

**Figure 12:**
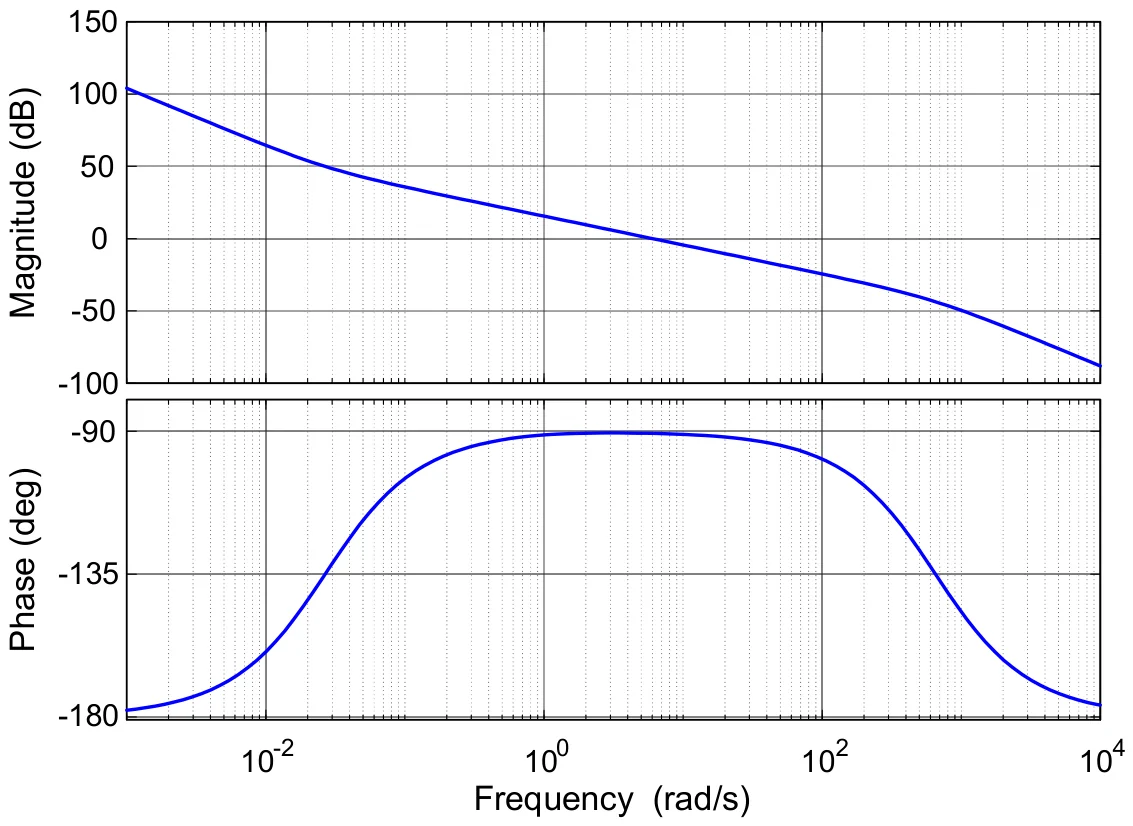
Bode plot of the open-loop system corresponding to the DIO-optimized PID-G controller.

For the linearized representation of the closed-loop system, stability is determined by the location of the roots of the closed-loop characteristic polynomial in the complex plane. The open-loop transfer function, obtained by cascading the linearized PID-G controller with the valve and actuator dynamics given in Equations (17) and (15), respectively, yields a rational transfer function whose gain crossover and phase crossover properties are directly reflected in the Bode diagram of Figure 12. The all-poles-in-left-half-plane condition for the closed-loop system is guaranteed when both the phase margin and the gain margin are strictly positive—a condition that is clearly satisfied by the results reported below. In this sense, the Bode analysis provides an indirect but rigorous verification of closed-loop stability for the linearized loop, and the boundedness of the Gompertz term provides additional assurance that this stability property is not fundamentally compromised by the nonlinear branch under practical operating conditions.

As shown in Figure 12, the magnitude response decreases continuously as the frequency increases, while the phase response exhibits the expected variation associated with the combined effect of the plant dynamics and the PID-G controller structure. The general shape of the Bode curves indicates a stable and well-behaved open-loop characteristic over the considered frequency range. In particular, the phase trajectory remains sufficiently separated from the critical −180° boundary around the gain crossover region, which is consistent with the favorable closed-loop behavior observed in the time-domain responses.

The main numerical stability indicators obtained from the Bode analysis further support this interpretation. The phase margin was found to be 89.4°, the delay margin was 0.258 s, and the peak gain was 784 dB. The phase margin of 89.4° indicates that the open-loop system can sustain an additional phase lag of nearly 90° at the gain crossover frequency before the onset of instability. This represents a substantial robustness reserve against dynamic perturbations, unmodeled actuator dynamics, and parameter variations—all of which are realistic sources of phase degradation in electro-hydraulic systems. From a theoretical standpoint, a phase margin of this magnitude implies that the closed-loop poles remain well inside the left half of the complex plane, corresponding to a strongly damped and non-oscillatory response character, which is fully consistent with the very low overshoot values obtained in the time-domain analysis. The delay margin of 0.258 s quantifies the maximum pure time delay that can be introduced into the feedback loop without causing instability. This metric is of direct practical relevance because implementation delays arising from signal conditioning, digital sampling, and communication latencies are unavoidable in real electro-hydraulic control systems. The fact that the DIO-optimized PID-G controller can tolerate up to 0.258 s of additional delay before destabilization confirms that the proposed design possesses a meaningful robustness buffer against such implementation imperfections. Together, the phase margin and the delay margin establish that the stability of the proposed closed-loop system is not marginal or sensitivity-dependent, but rather rests on a solid and quantifiable theoretical foundation. These margins carry direct implications for robustness beyond the nominal operating condition considered in the time-domain analyses. Because the phase and delay margins are derived from the same linearized plant and controller structure defined in Equations (15) and (17), they inherently reflect the closed-loop system’s tolerance to moderate variations in the underlying physical parameters, such as load mass, damping, spring stiffness, and pressure–flow characteristics, as well as to unmodeled dynamics and time delays of the kind associated with external load disturbances and component wear in electro-hydraulic systems. A systematic evaluation of the controller’s performance under explicitly imposed parameter perturbations and external disturbances, complementing the margin-based robustness indicators reported here, is identified in Section 7 as a direction for future investigation. The frequency-domain and time-domain findings therefore support one another in a mutually consistent manner: the controller not only achieves fast and accurate reference tracking, but also maintains a well-characterized and formally grounded stability reserve that is expressed through both the Bode indicators and the analytical boundedness property of the Gompertz nonlinearity. Taken as a whole, the theoretical treatment developed in this study establishes a coherent, control-theoretic account of how the Gompertz term influences both stability and dynamic performance, rather than describing its effect only through empirical tuning outcomes. The boundedness and monotonicity properties proven in Section 4 provide the analytical foundation for two complementary arguments developed elsewhere in this study: on the stability side, boundedness justifies treating the nonlinear branch as an additive perturbation on the linear PID loop, permitting the closed-loop stability margins to be assessed through the linearized open-loop analysis presented above; on the dynamic-performance side, the same boundedness and monotonicity properties explain, as discussed in Section 6.6, why the nonlinear term is able to accelerate the transient response during the large-error phase while withdrawing its contribution smoothly as the tracking error vanishes, without introducing additional overshoot. In this sense, the mechanism by which the Gompertz augmentation improves the closed-loop response is not treated as an incidental consequence of parameter tuning, but is derived directly from the analytical structure of the nonlinear term itself.

### 6.8. Setpoint Tracking and Controller Output

**Figure 13:**
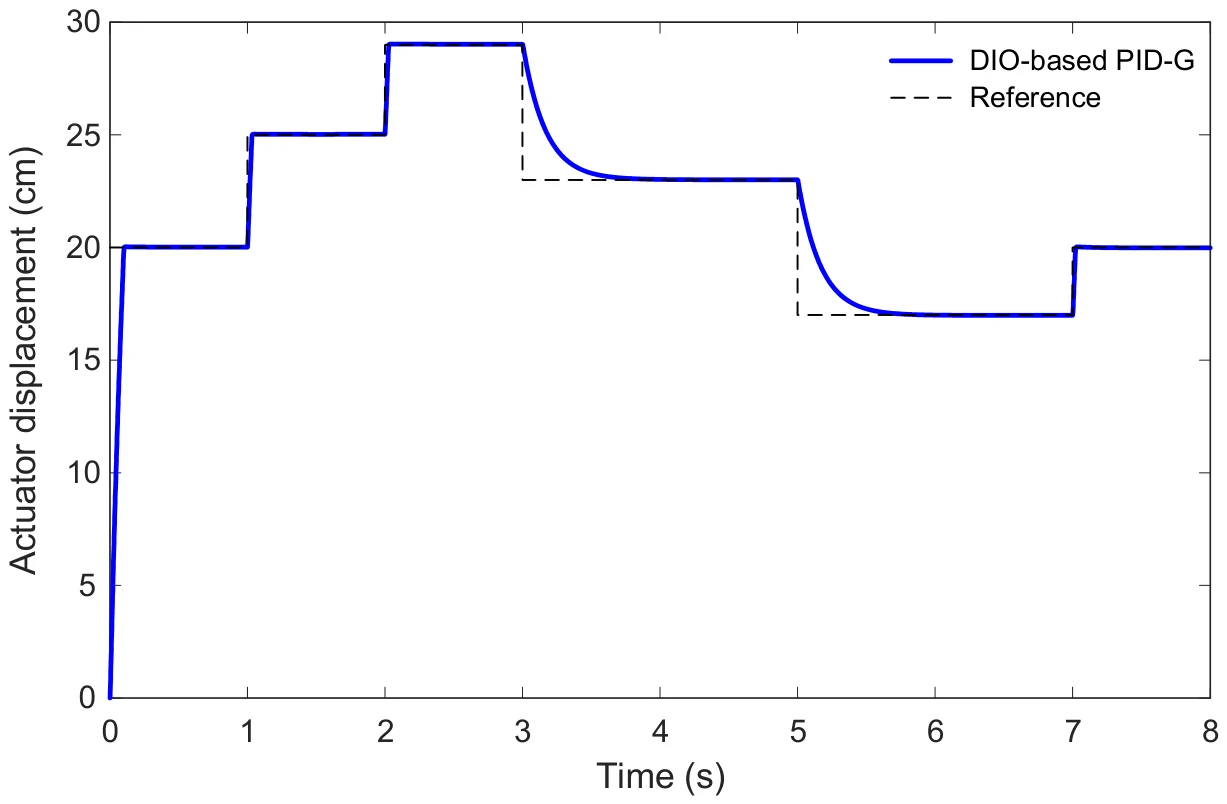
Setpoint-tracking performance of the DIO-optimized PID-G controller under varying displacement commands.

**Figure 14:**
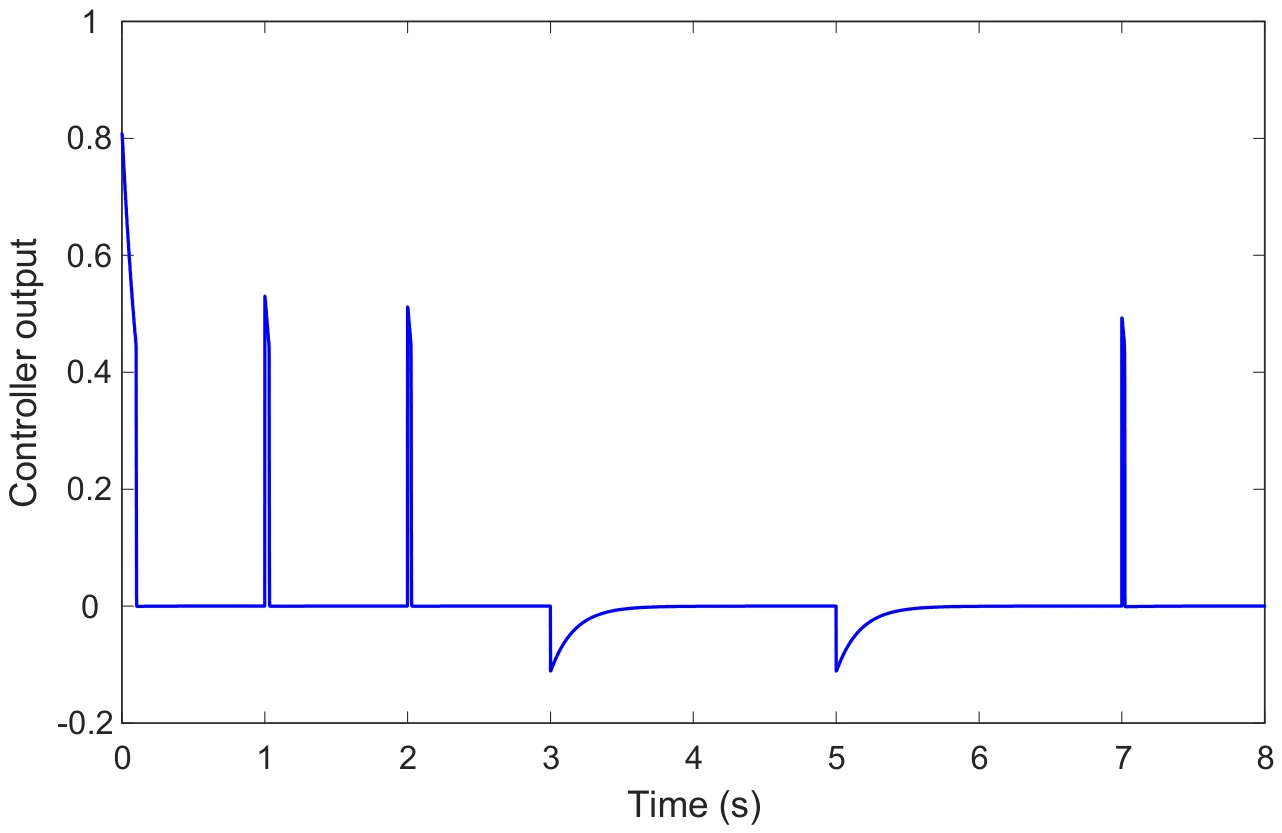
Controller output of the DIO-optimized PID-G controller corresponding to the varying setpoint-tracking test.

To further examine the practical tracking capability of the proposed controller beyond the single-step tests considered in the previous subsections, the DIO-optimized PID-G controller was evaluated under varying setpoint conditions. The resulting actuator displacement and controller output are presented in Figure 13 and Figure 14, respectively. This analysis is important because a controller designed for electro-hydraulic positioning should not only perform well for a fixed reference, but should also preserve accuracy and smoothness when the desired displacement changes over time. In this respect, the combined interpretation of the output trajectory and the control signal provides additional insight into the dynamic adaptability and practical realizability of the proposed control strategy. It should be noted that the quantitative transient indices for the DIO-optimized PID-G controller (rise time, settling time, overshoot, peak time, and steady-state error) have already been reported under the standardized single-step test in Table 4 (Section 6.4) and, in comparison with alternative PID-type structures, in Table 7 (Section 6.6). The present analysis serves a complementary purpose: rather than repeating this benchmarking exercise, it evaluates whether the favorable transient behavior established earlier is preserved when the reference changes repeatedly and by varying amounts, without returning to a common, settled initial condition between transitions. Because the six setpoint changes examined in Figure 13 differ from one another in both magnitude and direction, and because standard transient indices are defined relative to a step applied from a fixed initial state, tabulating separate numerical indices for each transition would not yield a directly comparable set of values in the manner that Table 4 provides across algorithms. The figures are therefore used here to convey the consistency of the qualitative response pattern (rapid, non-oscillatory convergence regardless of step size or direction) while the quantitative benchmarking of the controller’s transient performance remains anchored to the standardized tests reported in Section 6.4 and Section 6.6.

As shown in Figure 13, the reference signal is varied in a piecewise manner over the interval from 0 to 8 s. The actuator displacement follows these setpoint changes closely, with the output rapidly converging to each new command level. The initial increase toward 20 cm is achieved very quickly, which is consistent with the favorable transient performance reported earlier for the step-response case. Subsequent reference changes from 20 cm to 25 cm, from 25 cm to 29 cm, from 29 cm down to 23 cm, from 23 cm down to 17 cm, and finally from 17 cm back to 20 cm are also tracked successfully. The response remains smooth throughout these operating changes, and no sustained oscillatory behavior is observed. This indicates that the DIO-based PID-G controller preserves good tracking accuracy not only for a single operating point but also across multiple reference transitions. A particularly notable feature of Figure 13 is that the controller is able to accommodate both upward and downward changes in the desired displacement with a well-regulated response. During the upward transitions, the actuator displacement rises promptly to the new setpoint, whereas during the downward transitions the output decays in a controlled manner toward the lower command values. In both cases, the response remains close to the reference after a short transient interval. This behavior suggests that the nonlinear shaping introduced by the Gompertz function is beneficial not only for initial setpoint attainment, but also for repeated repositioning tasks in which the operating point varies over time.

The controller output shown in Figure 14 provides further support for this interpretation. It can be seen that the control signal exhibits short-duration peaks at the instants where the reference changes occur. Such peaks are expected, since a stronger control action is naturally required to drive the actuator rapidly toward a new position. After each transition, however, the control effort decreases and approaches a relatively small level, indicating that excessive sustained actuation is not required to maintain the commanded displacement. Negative excursions are observed during downward setpoint changes, which is also physically meaningful because the actuator must be driven in the opposite direction to follow the reduced reference level. Importantly, the control signal remains bounded throughout the entire test interval.

From a practical standpoint, the bounded nature of the controller output is an important result. Although transient peaks appear when the reference is changed, these peaks are localized and are not accompanied by erratic or highly oscillatory behavior. Therefore, the control action can be regarded as smooth and well-regulated in relation to the demanded tracking task. When considered together with the response in Figure 13, this result indicates that the proposed PID-G controller is capable of achieving accurate repositioning while maintaining a reasonable level of control effort.

### 6.9. Implementation Feasibility and Practical Considerations

Before discussing these considerations in detail, it is worth clarifying the rationale for adopting a simulation-based validation strategy at this stage of the work. Since the present study introduces both a new nonlinear controller structure and a new optimization algorithm for this specific electro-hydraulic control problem, the primary objective was to establish, through a systematically validated simulation framework, whether the proposed combination offers a consistent and genuine performance advantage over established alternatives before proceeding to hardware-in-the-loop or physical experimentation. This sequencing is consistent with common practice in controller-design research, where analytical and simulation-based validation typically precedes experimental confirmation once a new structural or optimization contribution has been shown to be theoretically sound. Accordingly, the present findings are not intended to substitute for physical validation, which, as noted in Section 7, remains the necessary subsequent step for capturing real-world effects such as friction, leakage, valve hysteresis, and sensor quantization that lie beyond the reach of any simulation model. Although the present study is conducted within a simulation environment, the results obtained across the various analyses collectively provide a meaningful basis for assessing the implementation feasibility of the proposed controller under real-world operating conditions. Three practical constraints of particular relevance to electro-hydraulic systems are considered here: measurement noise and signal quality, implementation delays, and actuator output saturation.

Regarding measurement noise, it is noted that the derivative channel of the PID-G controller incorporates a first-order low-pass filter governed by the coefficient N, which was included precisely to attenuate the high-frequency amplification that would otherwise render a pure differentiator impractical in sensor-based feedback loops. The DIO-optimized value of N=0.84914 corresponds to a relatively narrow derivative bandwidth, indicating that the optimizer naturally favored a well-filtered derivative action—a behavior that is consistent with what would be expected in a real implementation subject to transducer noise. This suggests that the proposed structure, as tuned, already embodies a degree of noise-awareness through its filtered derivative design.

With respect to implementation delays, the delay margin of 0.258 s reported in Section 6.7 provides direct quantitative evidence of the controller’s tolerance for pure time delay introduced by digital sampling, signal conditioning, and communication latency. In practical electro-hydraulic control systems, implementation delays are typically on the order of a few milliseconds. The 258 ms delay margin obtained for the DIO-optimized PID-G controller therefore represents a reserve that exceeds realistic implementation delays by approximately two orders of magnitude, indicating that the controller’s stability would not be compromised by the latencies encountered in standard embedded control hardware.

Concerning actuator output saturation, the controller output presented in Figure 14 demonstrates that the control signal remains bounded and well-regulated throughout the entire varying-setpoint test. The transient peaks observed at the instants of reference change are localized, moderate in amplitude, and followed by rapid decay to a low sustained level. This bounded and smooth behavior of the control signal suggests that the proposed PID-G controller, as tuned by DIO, does not generate excessively large output demands that would stress actuator capacity in a physical implementation. This property is partly attributable to the globally bounded nature of the Gompertz nonlinear term, which, as discussed in Section 6.7, satisfies 0<f(e)<a for all values of the tracking error, thereby preventing the nonlinear branch from contributing an unbounded output under any error condition. It is also relevant to note that the plant model adopted throughout this study, derived under the critically centered operating condition described in Section 3, represents a small-signal linearization of the underlying nonlinear orifice flow–pressure relationships governing the four-way valve. To assess the extent to which this simplification affects the reported conclusions, the closed-loop responses obtained under the linearized model were compared against those obtained under the corresponding nonlinear representation of the valve and actuator dynamics, retaining the nonlinear flow–pressure relationships prior to the linearization introduced in Equations (10)–(14). Over the displacement range and operating conditions examined in this study, including the varying-setpoint scenario of Section 6.8, the two representations produced closely consistent transient responses, indicating that the performance advantages reported for the DIO-optimized PID-G controller are not an artifact specific to the linearized formulation within this operating envelope. This comparison does not, however, substitute for validation under real-world nonidealities such as friction, leakage, and valve hysteresis, which fall outside the scope of a linearized or nonlinear simulation model alike and are appropriately addressed only through physical experimentation, as discussed below. Taken together, these observations indicate that the proposed DIO-based PID-G controller possesses several structural and performance characteristics that are favorable from an implementation standpoint, even in the absence of direct experimental confirmation. Experimental validation on a physical electro-hydraulic test rig, accounting explicitly for friction, leakage, valve hysteresis, and quantization effects, is identified as the primary direction for future work, as noted in the following section.

## 7. Conclusions

In this study, the displacement control problem of a four-way valve-controlled electro-hydraulic linear actuator has been addressed through the development of a PID controller augmented with a Gompertz function and tuned by the DIO. The work was built on a control-oriented mathematical model of the electro-hydraulic system, after which the proposed PID-G controller structure was formulated to combine the regulation capability of classical PID control with the nonlinear shaping effect of the Gompertz function. Since the resulting controller contains seven adjustable parameters, the tuning problem was expressed as an optimization task using a composite objective function that incorporates rise time, settling time, overshoot, and steady-state error in a unified manner. The simulation results have shown that DIO provides the most effective tuning performance among the considered optimization algorithms. When compared with FLA, CMA-ES, and PSO, DIO achieved the lowest objective-function levels, the most favorable statistical characteristics, and the best convergence behavior. These optimization advantages were directly reflected in the closed-loop response. The DIO-based PID-G controller yielded the best overall time-domain performance, with the shortest rise and settling times, very low overshoot, and the smallest steady-state error among the compared methods. In addition, the superiority of the DIO-based design was consistently confirmed through the integral error criteria IAE, ISE, ITAE, and ITSE, showing that the proposed approach reduces both the magnitude and persistence of the tracking error more effectively than the alternative algorithms. A further contribution of the study lies in the comparison of the proposed PID-G controller with several established PID-type controller variants, namely PI, PID, 2DOF-PID, and FOPID, all tuned under the same DIO framework. These results have demonstrated that the benefit of the proposed method is not limited to the optimizer alone. Rather, the nonlinear Gompertz augmentation provides an additional shaping capability that enables a more favorable balance between rapid response and tracking precision than that obtained by the benchmark controller structures. The PID-G controller outperformed all compared variants in both time-domain indices and error-based performance measures, indicating that the proposed nonlinear extension enhances the practical effectiveness of PID-based electro-hydraulic actuator control. The frequency-domain analysis further supported these findings by indicating a satisfactory stability reserve for the DIO-optimized PID-G controller. Moreover, the varying-setpoint tests showed that the proposed controller can track successive reference changes rapidly and smoothly while maintaining bounded control effort. Taken together, these results demonstrate that the proposed DIO-based PID-G design offers a coherent combination of effective optimization, strong transient performance, favorable tracking accuracy, and practically acceptable control behavior. Overall, the study demonstrates that the integration of DIO with a Gompertz-augmented PID structure provides a technically sound and effective control framework for electro-hydraulic actuator displacement systems. The findings suggest that this combined optimization and controller-design strategy can serve as a promising alternative to conventional PID-based approaches in applications where fast, accurate, and well-regulated positioning performance is required.

A balanced assessment of the proposed method requires that its limitations be acknowledged alongside its demonstrated advantages. Among the principal advantages, the PID-G structure provides a richer nonlinear shaping capability than conventional linear PID alternatives through the three independently adjustable Gompertz parameters, enabling a more favorable balance between transient speed and tracking precision without sacrificing smoothness near the setpoint. The globally bounded nature of the Gompertz term ensures that the nonlinear branch cannot generate unbounded control contributions, which is a desirable structural safety property. The DIO-based tuning framework offers a systematic and repeatable procedure for handling the resulting seven-parameter optimization problem, and the high phase and delay margins obtained confirm that the tuned controller possesses a substantial stability reserve. However, several limitations must also be recognized. The increase from three parameters in a standard PID to seven in the PID-G controller introduces a higher-dimensional search space, which demands a more capable optimizer and increases the computational effort of the offline tuning stage relative to simpler controller structures. The tuning procedure is entirely offline, meaning that the controller parameters remain fixed once the optimization is complete and no online adaptation is performed when plant conditions change. Furthermore, while the PID gains $K_{P}$, $K_{I}$, and $K_{D}$ carry clear physical interpretations related to error, accumulated error, and error rate, the Gompertz shape parameters a, b, and c are less intuitively linked to specific physical phenomena of the actuator, which may complicate manual re-tuning in practice. Beyond these considerations, the composite objective function of Equation (24) combines overshoot, steady-state error, rise time, and settling time through a single fixed weighting coefficient, φ=0.5, and the sensitivity of the reported optimum to this choice was not examined; a different weighting could, in principle, shift the relative emphasis placed on transient speed versus tracking accuracy and yield a different balance among the tuned parameters. In addition, the plant representation adopted in this study does not explicitly enforce actuator or valve saturation and rate limits beyond the intrinsic boundedness of the Gompertz nonlinear term, so the reported control signals, while shown to remain bounded and moderate in Section 6.9, have not been evaluated against explicit hardware-imposed actuation limits. The comparative evaluation is also limited in scope, in that it considers four metaheuristic optimizers and four PID-type controller structures; broader nonlinear or model-based control paradigms, such as sliding-mode or predictive control formulations, were not included in this comparison. Finally, all reported results correspond to a single nominal set of plant parameters, as listed in Table 1, and the sensitivity of the closed-loop performance to parametric uncertainty in the actuator and valve characteristics was not directly examined within the present study.

Notwithstanding the encouraging results obtained, several directions remain open for future investigation. First, the present study was conducted entirely within a simulation environment based on a linearized transfer-function model of the electro-hydraulic actuator. Experimental validation on a physical test rig, where real-world effects such as friction, leakage, sensor noise, and valve hysteresis are inevitably present, would constitute an important step toward confirming the practical applicability of the proposed design. Second, the mathematical model adopted in this study represents a linearized approximation of the actuator dynamics; future work could therefore examine the performance of the PID-G controller under more complete nonlinear plant descriptions, where the flow-pressure relationship and compressibility effects are modeled explicitly. Third, while the DIO-based tuning strategy was shown to be effective for the fixed operating conditions considered here, the extension of the framework to adaptive or gain-scheduled variants of the PID-G controller could be explored in order to address scenarios in which the plant parameters vary significantly over the operating range. Fourth, the robustness of the proposed controller under structured and unstructured uncertainties, external disturbances, and time-varying loads merits systematic investigation, potentially through the integration of uncertainty-aware objective functions or robust optimization strategies into the tuning procedure. A further useful extension would involve a systematic sensitivity analysis of the weighting coefficient adopted in the composite objective function of Equation (24), to establish how the relative emphasis placed on transient and steady-state performance indices influences the tuned parameter set and the resulting closed-loop behavior. Likewise, incorporating explicit actuator and valve saturation and rate-limit constraints into the plant model, together with an extended comparison against model-based or sliding-mode control formulations, would allow for the practical competitiveness of the proposed PID-G structure to be assessed under a broader and more demanding set of design conditions than those considered here. Finally, the Gompertz-augmented PID structure investigated in this study may be evaluated in the context of other hydraulic and mechatronic systems (such as electro-hydrostatic actuators, active suspension systems, or hydraulic manipulators) to assess the generalizability of the proposed approach beyond the four-way valve-controlled linear actuator considered here.

## Figures and Tables

**Table 1:** Parameters of the system adopted for this study.

Parameter	Value
Load damping ($D_{L}$)	1 N-s/m
Load spring stiffness ($k_{L}$)	1 N/m
Main spool area ($A_{m}$)	550 × 10^−6^ m^2^
Load mass (M)	1 kg
Actual piston area (A)	1.1 × 10^−3^ m^2^
Proportional control gain ($K_{a}$)	1
Main spool feedback transducer gain ($K_{f}$)	1
Flow gain of control valve ($K_{q}$)	0.359 m^2^/s
Pressure-flow coefficient ($K_{c}$)	1.7 × 10^−11^ m^3^/Pa/s
Ratio between input voltage and control spool displacement ($K_{r}$)	1
Transducer gain (h)	1

**Table 2:** Statistical results and nonparametric Wilcoxon test outcomes for the objective function values obtained by DIO, FLA, CMA-ES, and PSO over 30 independent runs.

Algorithm	Best	Worst	Average	Standard Deviation	*p*-Value
DIO	0.012965	0.014446	0.013465	0.00037145	---
FLA	0.014199	0.016048	0.014864	0.00041039	1.9209 × 10^−6^
CMA-ES	0.013637	0.015325	0.014152	0.00044505	2.8434 × 10^−5^
PSO	0.015139	0.017246	0.015845	0.00058489	1.7344 × 10^−6^

**Table 3:** Best PID-G controller parameters obtained by DIO, FLA, CMA-ES, and PSO.

Algorithm	$K_{P}$	$K_{I}$	$K_{D}$	N	a	b	c
DIO	0.018219	0.00048590	0.00035709	0.84914	0.43762	9.6039	13.2739
FLA	0.012856	0.00047729	0.00043565	0.90665	0.44063	7.0978	12.9815
CMA-ES	0.016533	0.00039138	0.00030794	0.63672	0.44103	8.3358	14.5317
PSO	0.018658	0.00051077	0.00038987	0.86785	0.35006	6.2050	14.3278

**Table 4:** Numerical time-domain performance comparison of the PID-G controllers optimized by DIO, FLA, CMA-ES, and PSO.

Algorithm	$t_{r}$ (s)	$t_{s}$ (s)	$M_{p}$ (cm)	OS (%)	$t_{p}$ (s)	$e_{ss}$ (%)
DIO	0.079511	0.099326	20.030	0.15110	0.1120	0.089317
FLA	0.085974	0.10699	20.049	0.24428	0.1190	0.12499
CMA-ES	0.081246	0.10135	20.055	0.27395	0.1130	0.092529
PSO	0.092410	0.11553	20.033	0.16560	0.1290	0.11749

**Table 5:** Performance of DIO, FLA, CMA-ES, and PSO in terms of minimizing IAE, ISE, ITAE and ITSE objective functions.

Algorithm	IAE (cm-s)	ISE (cm^2^-s)	ITAE (cm-s^2^)	ITSE (cm^2^-s^2^)
DIO	0.95041	12.053	0.038689	0.27922
FLA	1.0522	13.395	0.048435	0.34172
CMA-ES	0.98261	12.434	0.042261	0.29622
PSO	1.0828	13.555	0.050537	0.35838

**Table 6:** DIO-optimized parameters of PI, PID, 2DOF-PID, and FOPID controllers.

Controller	$K_{P}$	$K_{I}$	$K_{D}$	N	β	γ	λ	μ
PI	0.015029	0.00075804	-	-	-	-	-	-
PID	0.017017	0.00076748	0.00034232	0.89916	-	-	-	-
2DOF-PID	0.016946	0.00061310	0.00029819	1.2594	1.0094	1.0727	-	-
FOPID	0.019831	0.00068256	0.00027905	-	-	-	0.70513	0.76242

**Table 7:** Numerical time-domain performance comparison of the DIO-optimized PID-G, PI, PID, 2DOF-PID, and FOPID controllers.

Controller	$t_{r}$ (s)	$t_{s}$ (s)	$M_{p}$ (cm)	OS (%)	$t_{p}$ (s)	$e_{ss}$ (%)
PID-G	0.079511	0.099326	20.030	0.15110	0.1120	0.089317
PI	0.43161	0.71933	20.048	0.24223	1	0.24223
PID	0.37916	0.64803	20.053	0.26584	1	0.26584
2DOF-PID	0.36776	0.60358	20.205	1.0227	1	1.0227
FOPID	0.34096	0.59271	20.033	0.16685	1	0.16685

**Table 8:** Error-based performance comparison of the DIO-optimized PID-G, PI, PID, 2DOF-PID, and FOPID controllers in terms of IAE, ISE, ITAE, and ITSE.

Controller	IAE (cm-s)	ISE (cm^2^-s)	ITAE (cm-s^2^)	ITSE (cm^2^-s^2^)
PID-G	0.95041	12.053	0.038689	0.27922
PI	3.9298	40.71	0.71441	4.0169
PID	3.4576	35.512	0.56401	3.0702
2DOF-PID	3.3789	34.935	0.53921	2.9449
FOPID	2.9873	29.241	0.44770	2.2518

## Data Availability

All related data are available within the manuscript.
